# An aqueous molecular tube with polyaromatic frameworks capable of binding fluorescent dyes†
†Electronic supplementary information (ESI) available: Experimental procedures and physical data. See DOI: 10.1039/c4sc02377c


**DOI:** 10.1039/c4sc02377c

**Published:** 2014-09-12

**Authors:** Keita Hagiwara, Munetaka Akita, Michito Yoshizawa

**Affiliations:** a Chemical Resources Laboratory , Tokyo Institute of Technology , 4259 Nagatsuta, Midori-ku , Yokohama 226-8503 , Japan . Email: yoshizawa.m.ac@m.titech.ac.jp

## Abstract

An aqueous molecular tube composed of polyaromatic frameworks with peripheral hydrophilic groups was prepared. The new tube has a well-defined hydrophobic cavity with a diameter of ∼1 nm and quantitatively binds two molecules of fluorescent coumarin dyes in aqueous solutions. The bound coumarin dimers in a stacked fashion exhibit unusual excimer-like emissions in the confined space through efficient host–guest energy transfer.

## Abstract 2

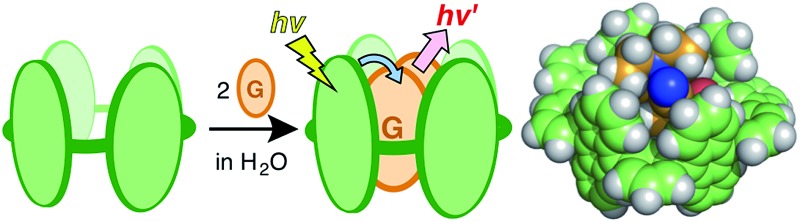
An aqueous polyaromatic tube binds two molecules of fluorescent dyes in water and the bound dye dimers exhibit unusual excimer-like emissions through efficient host–guest energy transfer.

## Introduction

**Fig. 1:**
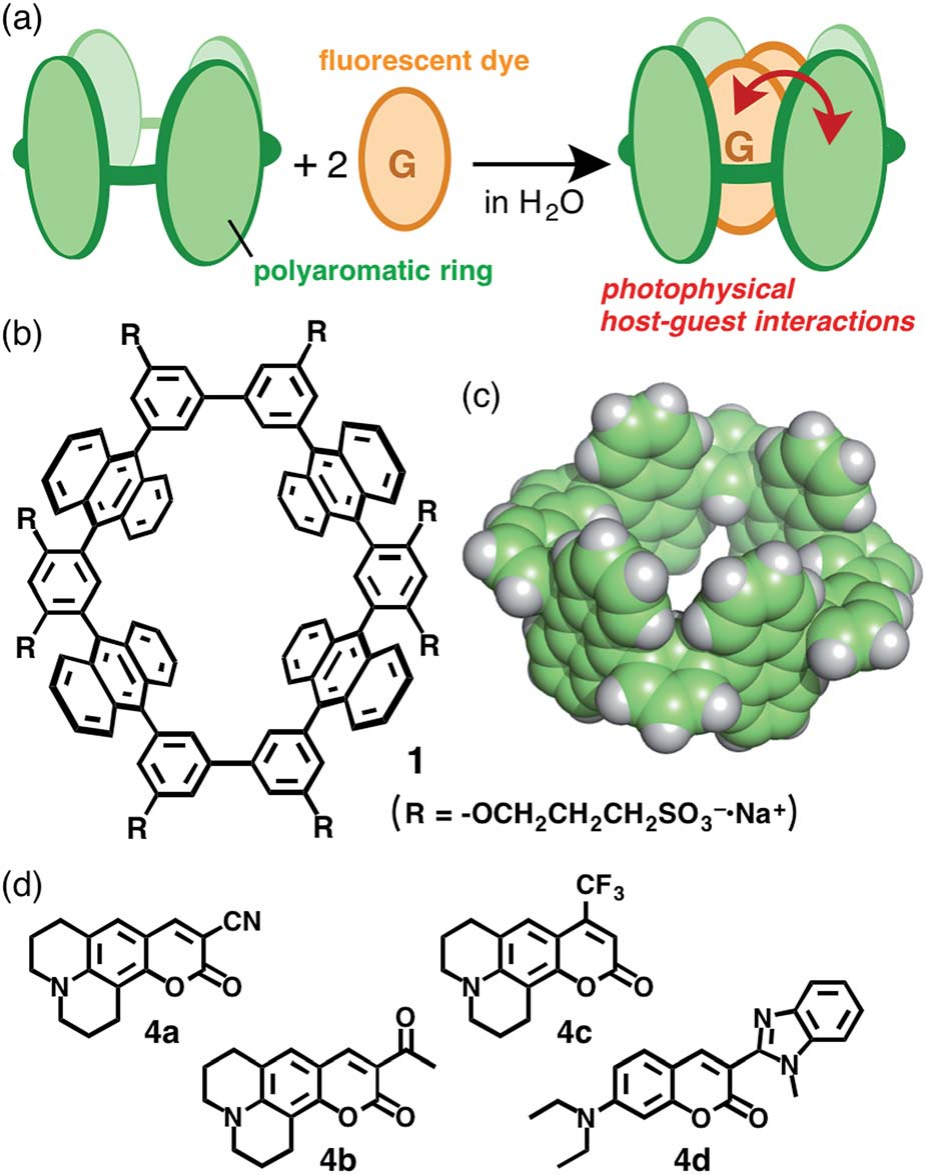
(a) Schematic representation of the encapsulation of guest molecules by an aqueous molecular tube with polyaromatic frameworks. (b) Aqueous molecular tube **1** and (c) the polyaromatic framework (R = –H). (d) Coumarin dyes **4a–d** employed as guest molecules.

Molecular rings and tubes composed of entirely aromatic frameworks, as contrasted with well-studied aliphatic ones (*e.g.*, cyclodextrins and cucurbiturils), have recently attracted greater attention from the viewpoint of both synthetic and physical chemistries due to their relevance to the partial structures of single-walled carbon nanotubes.^1,2^ Such aromatic macrocycles have been prepared by linking multiple benzene, naphthalene, pyrene, or chrysene rings^3,4^ through transition metal-mediated coupling reactions and they exhibit intriguing fluorescence properties based on their aromatic frameworks. However, their host–guest interactions have been limited to large spherical molecules, *i.e.* fullerenes,^5^ probably due to their large hydrophobic nature. Moreover, the supramolecular host–guest photo-phenomena of the aromatic macrocycles, as well as previous aliphatic tubes, have been relatively unexplored.^6,7^ We anticipated that the development of water-soluble molecular tubes with polyaromatic frameworks would lead to establishment of new photofunctional molecular flasks, providing wide-ranging host capabilities and unique host–guest photophysical properties (Fig. 1a). Here we present the preparation of a new aqueous molecular tube **1** with polyaromatic anthracene panels and peripheral hydrophilic sulfonate groups (R = –OCH_2_CH_2_CH_2_SO_3_^–^·Na^+^; Fig. 1b). The tube quantitatively binds two molecules of fluorescent coumarin dyes in aqueous solutions at room temperature. We emphasize that the bound coumarin dimers exhibit unusual excimer-like emissions in the confined tubular cavity through efficient energy transfer from the host framework to the guest dimers.

Recently, we have reported molecular tube **1′** (R = –OCH_3_ and –OCH_2_CH_2_OCH_3_) composed of four anthracene panels^8,9^ linked by phenylene and biphenylene spacers at the *meta* positions (Fig. 1b).^10^ The tubular structure has a well-defined 1 nm-sized cavity surrounded by hydrophobic anthracene frameworks (Fig. 1c), however its synthetic protocol demands a multistep preparation (15 steps; Scheme S1†) including a cross-coupling reaction between triflate-capped and boronate-capped half-tubes, which limits its further exploration. Besides, no host–guest interactions have been observed for the hydrophobic tube owing to its solubility in only organic solvents (*e.g.*, CHCl_3_ and acetone).

## Results and discussion

### Synthesis of an aqueous molecular tube

**Fig. 2:**
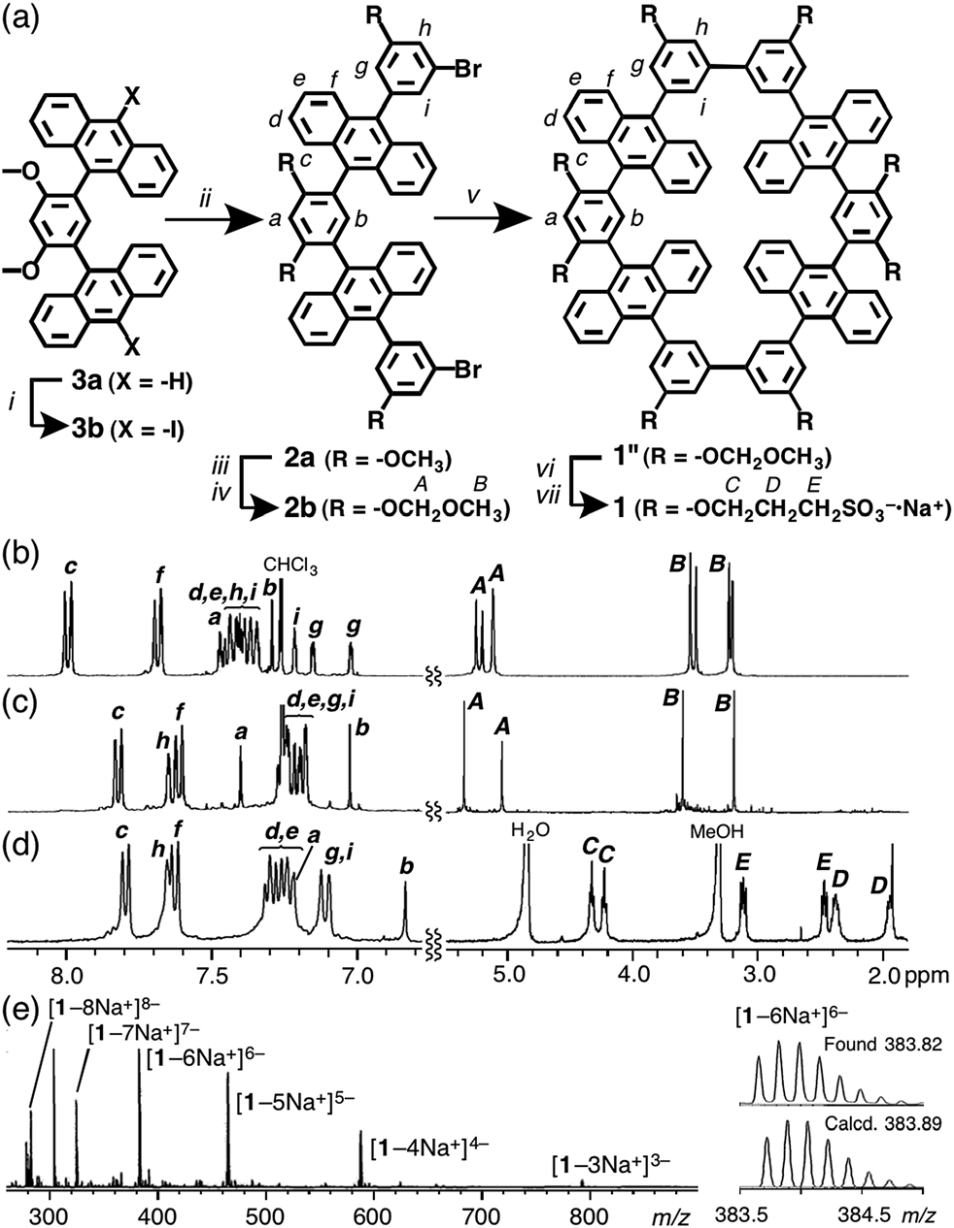
(a) Preparation of aqueous molecular tube **1**. Reagents and catalysts: (i) 1,3-diiodo-5,5-dimethylhydantoin; (ii) 3-bromo-5-methoxyphenylboronic acid pinacol ester, Pd(PPh_3_)_4_, Na_2_CO_3_; (iii) BBr_3_; (iv) chloromethyl methyl ether, Cs_2_CO_3_; (v) Ni(cod)_2_, 2,2′-bipyridyl; (vi) HCl_aq._; (vii) 1,3-propanesultone, NaH. ^1^H NMR spectra (400 MHz, r.t.) of (b) bromo-capped half-tube **2b** and (c) hydrophobic tube **1′′** in CDCl_3_, and (d) aqueous tube **1** in CD_3_OD. (e) ESI-TOF MS spectrum of aqueous tube **1** in CH_3_OH with an expansion and simulation of the [**1**–6Na^+^]^6–^ signal.

Water-soluble molecular tube **1** was obtained from anthracene dimer **3a** by the following straightforward synthetic route, which is 7-steps shorter than the previous route and includes bromo-capped half-tube **2a** as a key intermediate (Fig. 2 and ESI†). After the iodination of **3a**, the Suzuki–Miyaura coupling of **3b** (X = –I) with 3-bromo-5-methoxyphenyl boronate afforded **2a** in 54% yield. After the methoxy groups on **2a** were converted into 2-methoxymethoxy groups, a homo-coupling reaction of the methoxymethoxy-protected **2b** using a Ni(cod)_2_/bipyridine catalyst^11^ gave rise to tube **1′′** in a moderate yield (30%). Conversion of the peripheral substituents on **1′′** to hydrophilic sulfonate groups by sequential deprotection and etherification gave rise to aqueous tube **1** in 50% yield (2 steps).

The structures of molecular tubes **1** and half-tubes **2** were fully confirmed by NMR spectroscopy and mass spectrometry.† The ^1^H NMR spectrum of tube precursor **2b** (Fig. 2b) showed the corresponding aromatic H_a–i_ and aliphatic H_A,B_ signals as assigned by COSY and NOESY analyses. Characteristically, two sets of proton signals derived from the functionalized phenyl moieties (H_g,i_ and H_A,B_) were observed in a 1 : 1 ratio.^12^ On the other hand, the ^1^H NMR analysis of **1′′** showed a simple spectrum displaying nine aromatic and four aliphatic signals (Fig. 2c). This spectral change is reasonable upon the formation of tubular compound **1′′** with *D*_2h_ symmetry. Matrix-assisted laser desorption ionization time-of-flight mass spectrometry (MALDI-TOF MS) revealed the formation of **1′′** with a prominent peak at *m*/*z* = 1641.44. After isolation of aqueous tube **1**, the ^1^H NMR spectrum in CD_3_OD exhibited new aliphatic signals attributed to the decorated sulfonate groups (H_C–E_) in the range 4.4–1.8 ppm (Fig. 2d). Electrospray ionization (ESI)-TOF MS spectrum of **1** showed a series of intense peaks at *e.g.*, 791.0 [**1**–3Na^+^]^3–^, 587.2 [**1**–4Na^+^]^4–^, and 465.2 [**1**–5Na^+^]^5–^ (Fig. 2e), which unambiguously elucidated full modification of the tube by the hydrophilic groups.

**Fig. 3:**
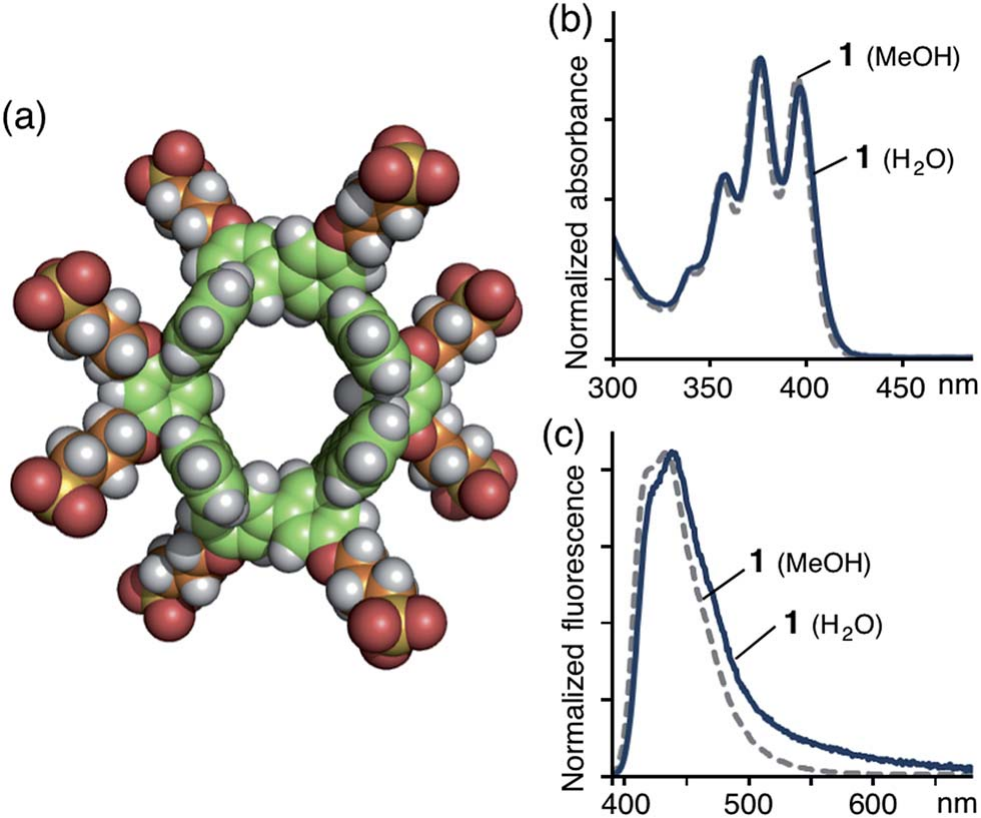
(a) An optimized structure of aqueous tube **1** without counterions or solvents by PM3 calculation. (b) UV-vis spectra (10 μM, r.t.) and (c) fluorescence spectra (*λ*_ex_ = 378 nm, 10 μM, r.t.) of **1** in H_2_O (solid line) and CH_3_OH (dashed line).

Obtained sulfonate-coated tube **1** is highly soluble in water (>100 mM) but tubes **1′** and **1′′** are effectively insoluble in water. In the ^1^H NMR spectrum of **1** in D_2_O, the proton signals were significantly broadened (Fig. S20†), most probably originating from slow intramolecular motion on the NMR timescale. Dynamic light scattering (DLS) analysis of aqueous solutions of **1** showed small particles with an average diameter of 1.4 nm (Fig. S21a†), which indicates no aggregation of **1** in water. An optimized structure of **1** shows that its external and core diameters are approximately 1.9 and 1.0 nm, respectively (Fig. 3a).

The UV-vis spectrum of tube **1** in H_2_O exhibited the typical π–π* transition bands of the anthracene moieties (Fig. 3b). The emission spectra of **1** in H_2_O and CH_3_OH displayed a broad band in the range 390–550 nm (*λ*_max_ = ∼440 nm; Fig. 3c) with good absolute quantum yields (*Φ*_F_ = 30 and 59%, respectively).† Thus, the obtained polyaromatic nanotube providing both good water-solubility and fluorescence properties would be expected to act as a photofunctional molecular host in aqueous media.

### Encapsulation of coumarin dyes

**Fig. 4:**
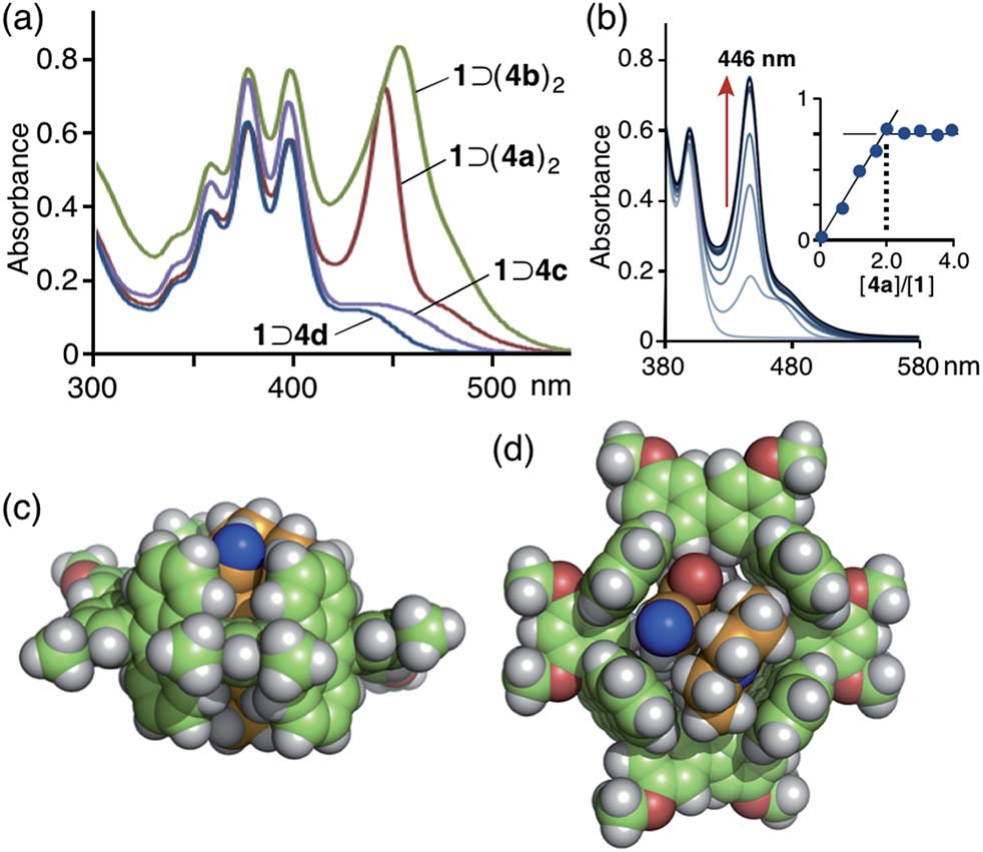
(a) UV-vis spectra (0.2 mM, H_2_O, r.t.) of **1** ⊃ (**4a**)_2_, **1** ⊃ (**4b**)_2_, **1** ⊃ **4c**, and **1** ⊃ **4d**. (b) Titration UV-vis spectra (0.2 mM, H_2_O, r.t.) and the plot (*λ*_abs_ = 446 nm) of **1** with the addition of **4a** ([**4a**]/[**1**] = 0.5, 1.0, 1.5, 2.0, 2.5, 3.0, 3.5, 4.0). Optimized structure of **1** ⊃ (**4a**)_2_ (R = –OCH_3_): (c) side and (d) top views.

The open-ended and 1 nm-length cylindrical cavity of tube **1** facilitated the quantitative encapsulation of two molecules of coumarin 337 (**4a**) and 334 (**4b**), well-known hydrophobic fluorescent dyes, in aqueous solutions through hydrophobic and aromatic–aromatic interactions (Fig. 1a and d). When a slight excess of 3-cyano-substituted **4a** was suspended in an aqueous solution (0.2 mM) of tube **1** at room temperature for 1 h, the color of the solution obviously changed from colorless to yellow due to the formation of a 1 : 2 host–guest complex, **1** ⊃ (**4a**)_2_, as revealed by UV-vis and MS analyses.^13^ The UV-vis absorption spectrum of the product showed a new prominent band at *λ*_max_ = 446 nm corresponding to the bound coumarin guests (Fig. 4a). The band is considerably narrower than that of free **4a** in CH_3_OH (Fig. S24†). It is worthy of note that another new absorption band was observed as a shoulder at around 480 nm, which can presumably be attributed to a π-stacked coumarin dimer. In combination with ESI-TOF MS analysis,^14^ a UV-vis spectral titration study clearly illustrated the quantitative formation of the 1 : 2 host–guest structure (Fig. 4b).† Similarly, two molecules of 3-acetyl-substituted coumarin **4b** were quantitatively bound in the cavity of tube **1** to produce **1** ⊃ (**4b**)_2_, which showed an absorption band for the guests at *λ*_max_ = 455 nm (Fig. 4a). Notably, the absorption bands of **4a** and **4b** overlap considerably with the emission band of **1** (Fig. 3c). An optimized structure of **1** ⊃(**4a**)_2_ (R = –OCH_3_) obtained by force-field calculations^13,15^ indicated that the two coumarin guests adopt a π-stacked *anti*-head-to-tail conformation with an interplanar distance of 3.5 Å in the cavity (Fig. 4c and d). The stacked dimer fully occupies the nanospace and is in tight contact with the four anthracene panels of the tube.

Interestingly, in the case of 4-trifluoromethyl-substituted coumarin 153 (**4c**) and 3-(*N*-methyl)benzimidazolyl-substituted coumarin 30 (**4d**), tube **1** recognized the substituents on the coumarin dyes and yielded 1 : 1 host–guest complexes.^10^ UV-vis and ESI-TOF MS studies revealed the formation of **1** ⊃ **4c** and **1** ⊃ **4d** (in 40 and 30% yields, respectively) under conditions similar to those used for **1** ⊃ (**4a**)_2_. The UV-vis spectra showed new absorption bands resulting from bound coumarins **4c** and **4d** at *λ*_max_ = 437 and 430 nm, respectively (Fig. 4a). Force-field calculation studies suggested that the 4-trifluoromethyl and *N*-methylbenzimidazolyl groups on the coumarin dyes prevent the formation of 1 : 2 host–guest structures and efficient host–guest stacking interactions due to steric hindrance (Fig. S34 and S38†). The competitive binding experiment for tube **1** revealed the preferential binding series of **4a** ≈ **4b** ≫ **4c** ≈ **4d** for the coumarin guests.†

### Unique host–guest photophysical properties

**Fig. 5:**
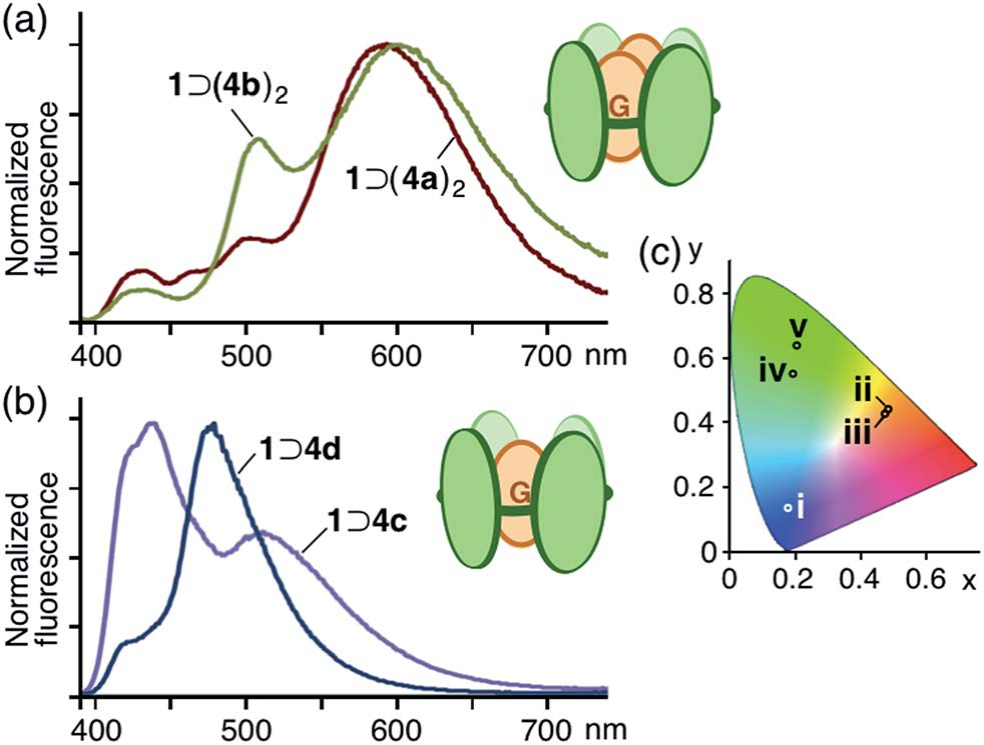
Fluorescence spectra (*λ*_ex_ = 378 nm, H_2_O, r.t.) of (a) the 1 : 2 host–guest complexes and (b) the 1 : 1 host–guest complexes. (c) CIE chromaticity diagram of the fluorescence color of (i) **1**, (ii) **1** ⊃ (**4a**)_2_, (iii) **1** ⊃ (**4b**)_2_ in H_2_O and (iv) **4a**, (v) **4b** in CH_3_OH.

The aqueous solutions of 1 : 2 host–guest complexes **1** ⊃ (**4a**)_2_ and **1** ⊃ (**4b**)_2_ exhibited unusual emissions from the stacked coumarin dimers upon irradiation of the host framework at 378 nm at room temperature. The fluorescence spectrum of **1** ⊃ (**4a**)_2_ contained a new broad band at around 590 nm, assignable to an excimer-like emission from (**4a**)_2_ within **1**,^16^ whereas emission from the anthracene moieties of **1** (∼440 nm) and the monomeric coumarin guest (∼510 nm) were markedly weaker (Fig. 5a) due to efficient fluorescence resonance energy transfer (FRET)^17^ from host **1** to guest (**4a**)_2_. Similarly, **1** ⊃ (**4b**)_2_ showed an excimer-like emission band at around 600 nm in the fluorescence spectrum, accompanying a weak emission band (∼500 nm) for the monomeric coumarin guest. In sharp contrast, the aqueous solution of a mixture of **1** ⊃ **4c** and **1** displayed only host and guest emissions (∼440 and ∼510 nm, respectively) and a mixture of **1** ⊃ **4d** and **1** displayed a prominent guest emission at *λ*_max_ = 477 nm through FRET upon irradiation at 378 nm (Fig. 5b). The CIE chromaticity diagram for **1** ⊃ (**4a**)_2_ and **1** ⊃ (**4b**)_2_ quantified the emission colors as orange ((0.48, 0.44) and (0.48, 0.43), respectively), unlike that for **1**, **4a**, and **4b** (Fig. 5c).

The water-solubility of tube **1** is essential to both the strong binding of the hydrophobic fluorescent dyes and the unique emissions of the bound dyes through efficient host–guest and guest–guest interactions. When **1** ⊃ (**4a**)_2_ was dissolved in MeOH, the host–guest complex dissociated immediately. In the fluorescence spectrum, only emission bands from free **1** and **4a** were observed upon irradiation at 378 nm (Fig. S24†).

## Conclusions

In conclusion, we have succeeded in the preparation of a molecular tube with polyaromatic panels and peripheral hydrophilic groups. The new aqueous tube quantitatively binds two molecules of fluorescent coumarin dyes in its well-defined nanospace through hydrophobic and aromatic–aromatic interactions in water. Notably and most importantly, the bound fluorescent dyes in a stacked fashion exhibited excimer-like emission through efficient host–guest intermolecular interactions. This study demonstrates that photoactive and water-soluble polyaromatic tubes have potential for exploring novel host–guest supramolecular structures and subsequent unique photophysical properties.
