# Supplementary material for: An aqueous molecular tube with polyaromatic frameworks capable of binding fluorescent dyes
Source: Chem Sci. 2014 Sep 12;6(1):259–63. doi: 10.1039/c4sc02377c (PMC5435871; doi:10.1039/c4sc02377c)
Supplement: Supplementary file 1 [file SC-006-C4SC02377C-s001.pdf]

Electronic Supplementary Material (ESI) for Chemical Science.
This journal is © The Royal Society of Chemistry 2014

Electronic Supplementary Information

An Aqueous Molecular Tube with Polyaromatic Frameworks Capable of Binding Fluorescent Dyes

Keita Hagiwara, Munetaka Akita, Michito Yoshizawa*

Chemical Resources Laboratory, Tokyo Institute of Technology, 4259 Nagatsuta, Midori-ku, Yokohama 226-8503, Japan

Fax: (+81)45-924-5230, E-mail: yoshizawa.m.ac@m.titech.ac.jp

Contents

- Materials and methods
- Synthetic routes of tubes **1'** and **1"**
- Synthesis of 3-bromo-5-methoxyphenylboronic acid pinacol ester
- Synthesis of anthracene dimer **3b**
- Synthesis of half-tube **2a** (^{1}H and ^{13}C NMR, ^{1}H-^{1}H COSY, HSQC, MALDI-TOF MS spectra)
- Synthesis of half-tube **2b** (^{1}H and ^{13}C NMR, MALDI-TOF MS spectra)
- Synthesis of tube **1"** (^{1}H NMR and MALDI-TOF MS spectra)
- Synthesis of tube **1** (^{1}H and ^{13}C NMR, HSQC, ESI-TOF MS, UV-vis, fluorescence spectra)
- Synthesis and properties of **1**⊃(**4a**)$_2$ (^{1}H NMR, UV-vis, fluorescence, ESI-TOF MS spectra, and DLS, fluorescence lifetime, optimized structure)
- Synthesis and properties of **1**⊃(**4b**)$_2$ (UV-vis, fluorescence, ESI-TOF MS spectra, and optimized structure)
- Synthesis and properties of **1**⊃**4c** (UV-vis, fluorescence, ESI-TOF MS spectra, and optimized structure)
- Synthesis and properties of **1**⊃**4d** (UV-vis, fluorescence, ESI-TOF MS spectra, and optimized structure)
- CIE diagram of tube **1** and host-guest complexes
- Competitive binding experiment of coumarin guests

Materials and methods

NMR: Bruker AVANCE-400 (400 MHz), GC MS: Shimadzu Parvum2/ULBON HR-1, MALDI-TOF MS: Shimadzu AXIMA-CFR Plus, ESI-TOF MS: Bruker micrOTOF II, FT IR: JASCO FT/IR-4200, UV-vis: JASCO V-670DS, Fluorescence: SHIMADZU RF-5300PC, Elemental analysis: LECO CHNS-932 VTF-900, Absolute PL quantum yield: Hamamatsu C9920-02G with an integration sphere, Fluorescence lifetime: Hamamatsu C11367-01, Recycled GPC: JAI LC-9225NEXT, DLS: Wyatt Technology DynaPro NanoStar, Force-field calculation: Materials Studio version 5.0 (Accelrys Software Inc., San Diego, CA).

Solvents and reagents: TCI Co., Ltd., WAKO Pure Chemical Industries Ltd., KANTO CHEMICAL CO., INC., Sigma-Aldrich Co., and Cambridge Isotope Laboratories, Inc. Anthracene dimers **3a** and **3b** were synthesized according to previously reported procedures (M. Yoshizawa *et al*., *J. Am. Chem. Soc*., **2011**, *133*, 11438–11441 and *Chem. Asian J.,* **2014**, *9*, 1016–1019).

Scheme S1. Previous synthetic route of tube **1’**.

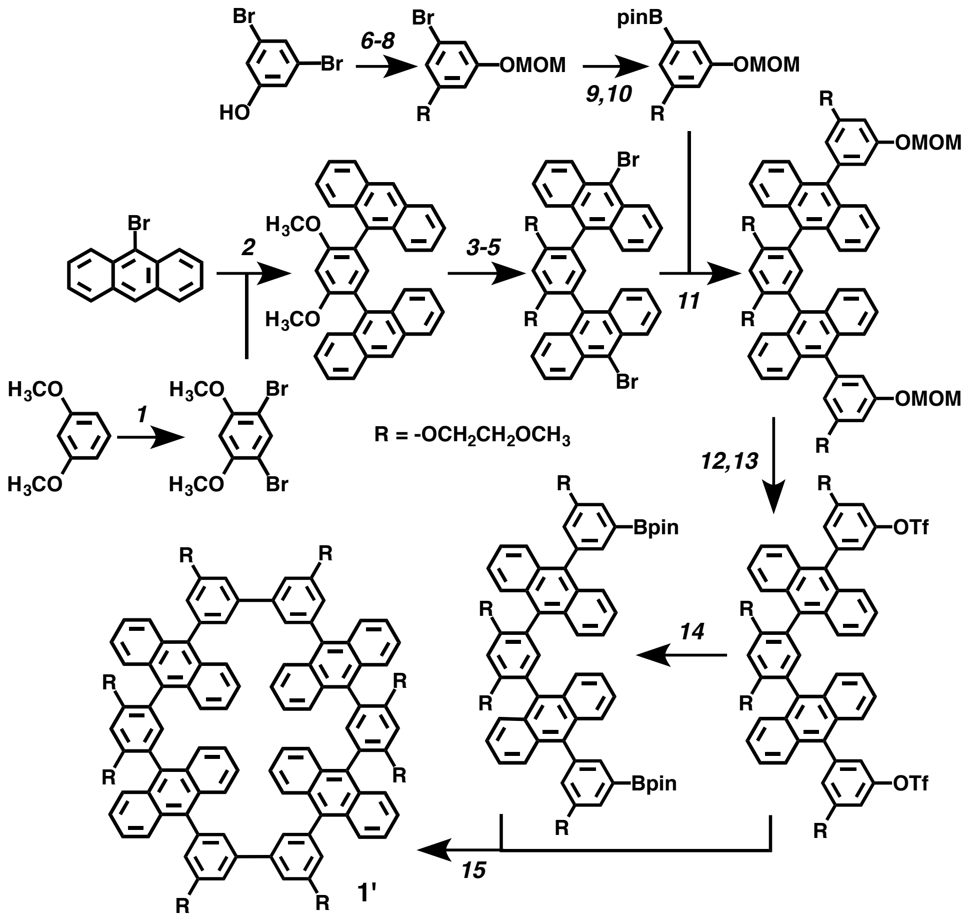

Scheme S2. New synthetic route of tube **1”**.

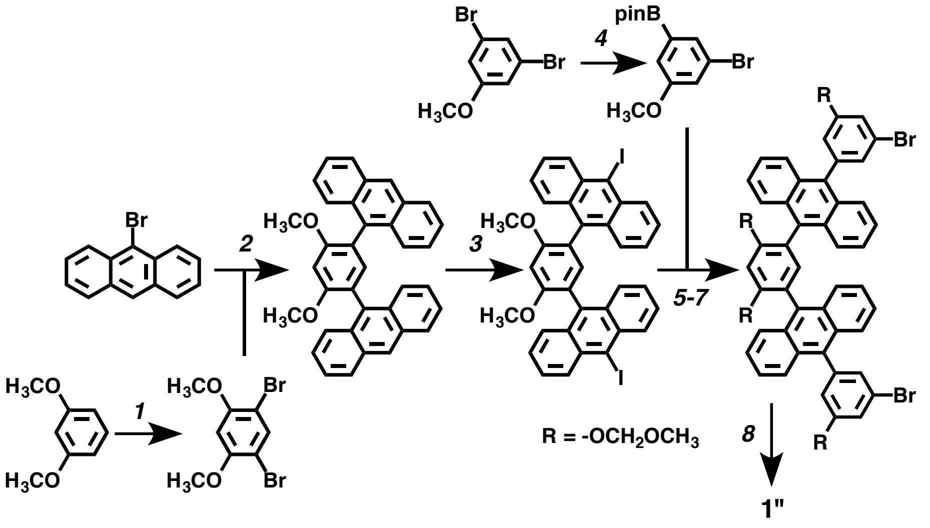

Synthesis of 3-bromo-5-methoxyphenylboronic acid pinacol ester

KH-268, (283, 296)

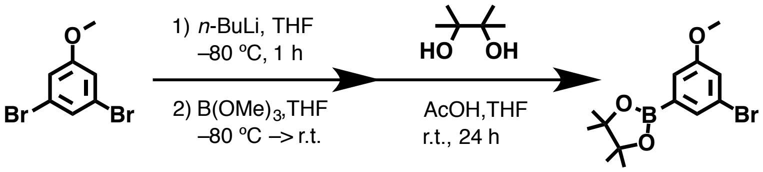

1,3-Dibromo-5-methoxybenzene (2.010 g, 7.558 mmol) and dry THF (100 mL) were added to a 2-necked 200 mL glass flask filled with N_2. A hexane solution (2.69 M) of *n*-butyllithium (3.0 mL, 7.8 mmol) was then added dropwise to this flask at –80 ºC under N_2. After the mixture was stirred at –80 ºC for 1 h, a dry THF solution (5 mL) of $B(OCH_3)_3$ (1.0 mL, 9.0 mmol) was added to the solution. The resultant mixture was further stirred at –80 ºC for 1 h and then warmed to r.t. for 1 h. Pinacol (1.280 g, 1.083 mmol) and AcOH (1 mL) were added to the solution and the resultant solution was stirred at r.t. for 24 h. The products were extracted with CH_2Cl_2 and the combined organic phase was dried over $MgSO_4$, filtrated, and concentrated under reduced pressure. The crude product was purified by silica-gel column chromatography (hexane:ethyl acetate = 10:1) to afford 3-bromo-5-methoxyphenylboronic acid pinacol ester as a yellow solution (1.975 g, 6.310 mmol, 83%).

^{1}H NMR (400 MHz, $CDCl_3$, r.t.): δ 7.52 (d, J = 2.0 Hz, 1H), 7.24 (d, J = 2.0 Hz, 1H), 7.15 (dd, J = 2.0, 2.0 Hz, 1H), 3.81 (s, 3H), 1.34 (s, 12H). GC-MS: m/z Calcd. for $C_{13}H_{18}BBrO_3$ 312, Found 312 $[M]^+$.

Synthesis of anthracene dimer 3b KH-287, (267, 283)

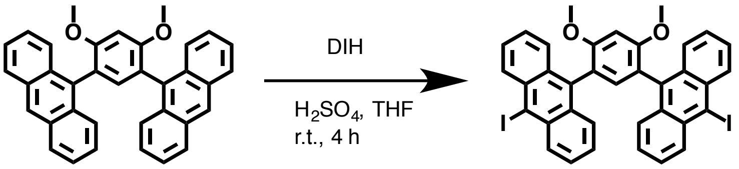

Anthracene dimer **3a** (7.762 g, 15.82 mmol) and THF (100 mL) were added to a 200 mL glass flask. 1,3-Diiodo-5,5-dimethylhydantoin (DIH; 8.036 g, 20.43 mmol) was added to the solution at 0 ºC and then concentrated H_2SO_4 (0.5 mL) was added to the solution. The resultant mixture was stirred at r.t. for 1 d. A precipitated crude product was washed with CH_3OH, H_2O, and hexane to afford **3b** as a yellow solid (7.679 g, 10.34 mmol; 65%).

^{1}H NMR (400 MHz, $CDCl_3$, r.t.): δ 8.54 (d, J = 8.8 Hz, 4H), 7.86 (d, J = 8.8 Hz, 4H), 7.57-7.52 (m, 4H), 7.46-7.43 (m, 4H), 7.15 (s, 1H), 6.97 (s, 1H), 3.78 (s, 6H). ^{13}C NMR (100 MHz, $CDCl_3$, r.t.): δ 159.1 (C_q), 136.5 (CH), 135.5 (C_q), 134.0 (CH), 133.8 (C_q), 131.7 (C_q), 127.6 (CH), 127.5 (CH), 125.8 (CH), 119.2 (C_q), 105.9 (C_q), 96.3 (CH), 56.2 (CH_3). FT-IR (KBr, cm^{-1}): 3440, 3068, 2942, 2836, 1606, 1506, 1450, 1330, 1261, 1201, 1157, 1029, 866, 752. MALDI-TOF MS (dithranol): m/z Calcd. for $C_{36}H_{24}I_2O_2$ $[M]^+$ 741.99, Found 741.80.

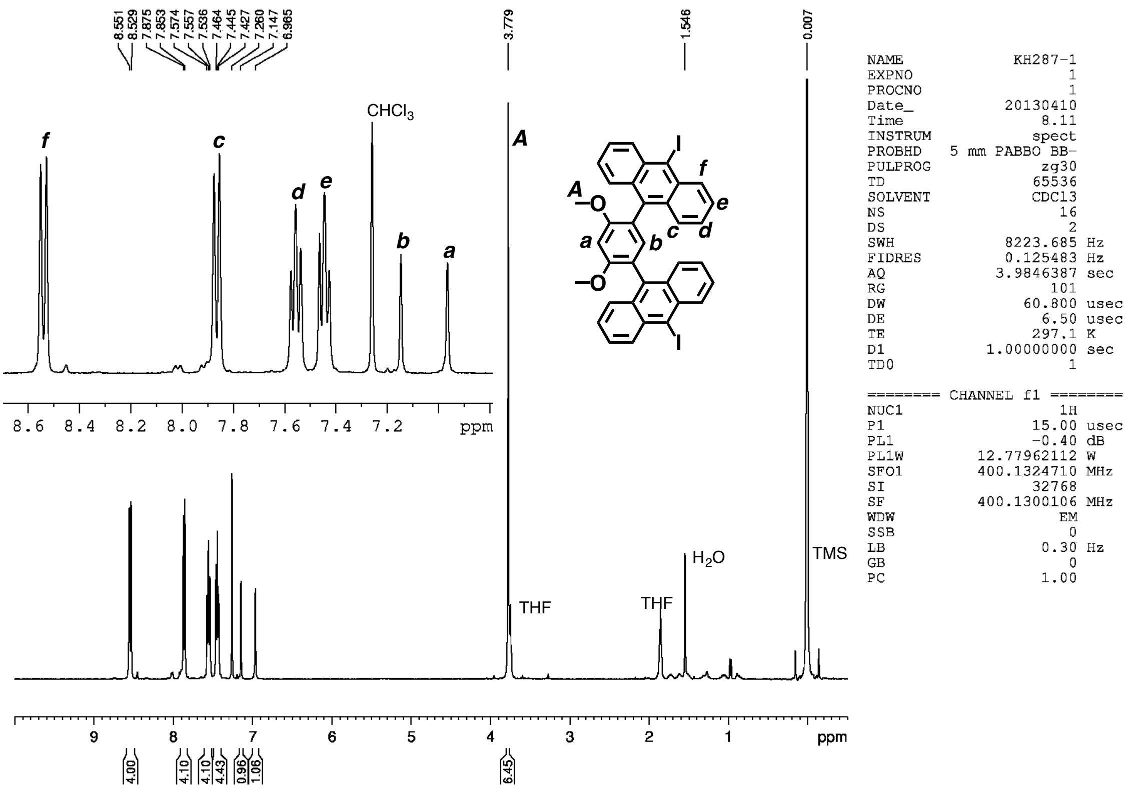

Figure S1. ^{1}H NMR spectrum (400 MHz, $CDCl_3$, r.t.) of **3b**.

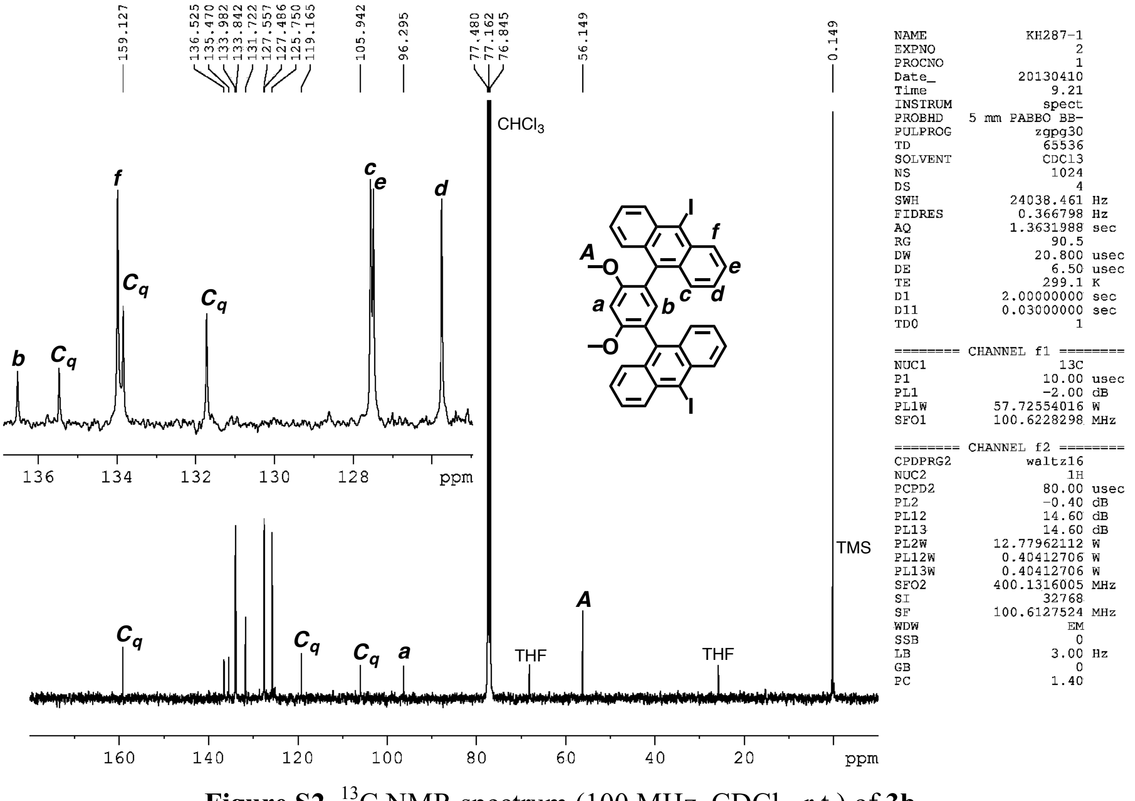

Figure S2. ^{13}C NMR spectrum (100 MHz, $CDCl_3$, r.t.) of **3b**.

Synthesis of half-tube 2a KH-270, (286)

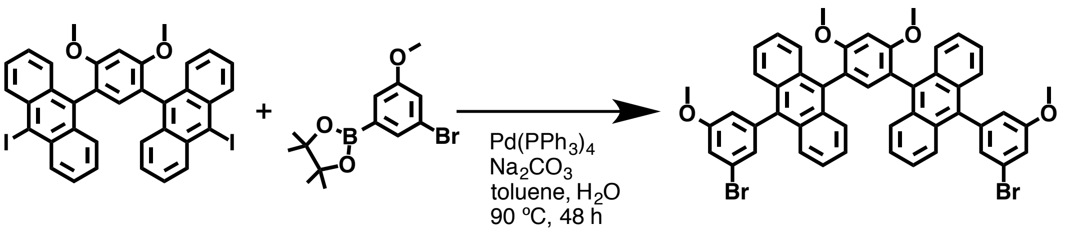

Anthracene dimer **3b** (1.952 g, 2.629 mmol), 3-bromo-5-methoxyphenylboronic acid pinacol ester (1.975 g, 6.310 mmol), $Pd(PPh_3)_4$ (0.154 g, 0.133 mmol), and toluene (150 mL) were added to a 2-necked 100 mL glass flask filled with N_2. A degassed aqueous solution (25 mL) of Na_2CO_3 (3.824 g, 36.07 mmol) was added to this flask and the resultant mixture was stirred at 100 ºC for 48 h. The mixture was concentrated under reduce pressure and the crude product was extracted with $CHCl_3$. The obtained crude product was purified by silica-gel column chromatography (hexane:ethyl acetate = 10:1) and GPC ($CHCl_3$) to afford half-tube **2a** as a yellow solid (1.233 g, 1.432 mmol; 54%).
^{1}H NMR (400 MHz, $CDCl_3$, r.t.): δ 7.97 (d, J = 8.8 Hz, 4H), 7.68 (d, J = 8.8 Hz, 4H), 7.43 (dd, J = 8.8, 7.6 Hz, 4H), 7.36 (dd, J = 8.8, 7.6 Hz, 4H), 7.29 (s, 1H), 7.26 (s, 1H, overlapped by $CHCl_3$), 7.24 (s, 2H), 7.16 (s, 1H), 7.03-7.02 (m, 2H), 6.89 (s, 1H), 3.86 (s, 3H), 3.85 (s, 3H), 3.84 (s, 3H), 3.81 (s, 3H). ^{13}C NMR (100 MHz, $CDCl_3$, r.t.): δ 160.4 (C_q), 159.1 (C_q), 142.4 (C_q), 137.1 (CH), 135.2 (C_q), 134.1 (C_q), 130.5 (C_q), 129.9 (C_q), 127.1 (CH), 126.9 (CH), 126.8 (CH), 125.4 (CH), 125.2 (CH), 123.0 (C_q), 119.5 (C_q), 116.7 (CH), 116.1 (CH), 96.3 (CH), 56.2 (CH_3), 55.8 (CH_3). FT-IR (KBr, cm^{-1}): 3068, 3007, 2941, 2837, 1597, 1454, 1371, 1259, 1201, 1041, 847, 766. MALDI-TOF MS (dithranol): m/z Calcd. for $C_{50}H_{36}Br_2O_4$ $[M]^+$ 860.10, Found 859.88. E.A.: Calcd. for $C_{50}H_{36}Br_2O_4$•$0.5CH_2Cl_2$: C, 67.16; H, 4.13. Found: C, 67.27; H, 4.00.

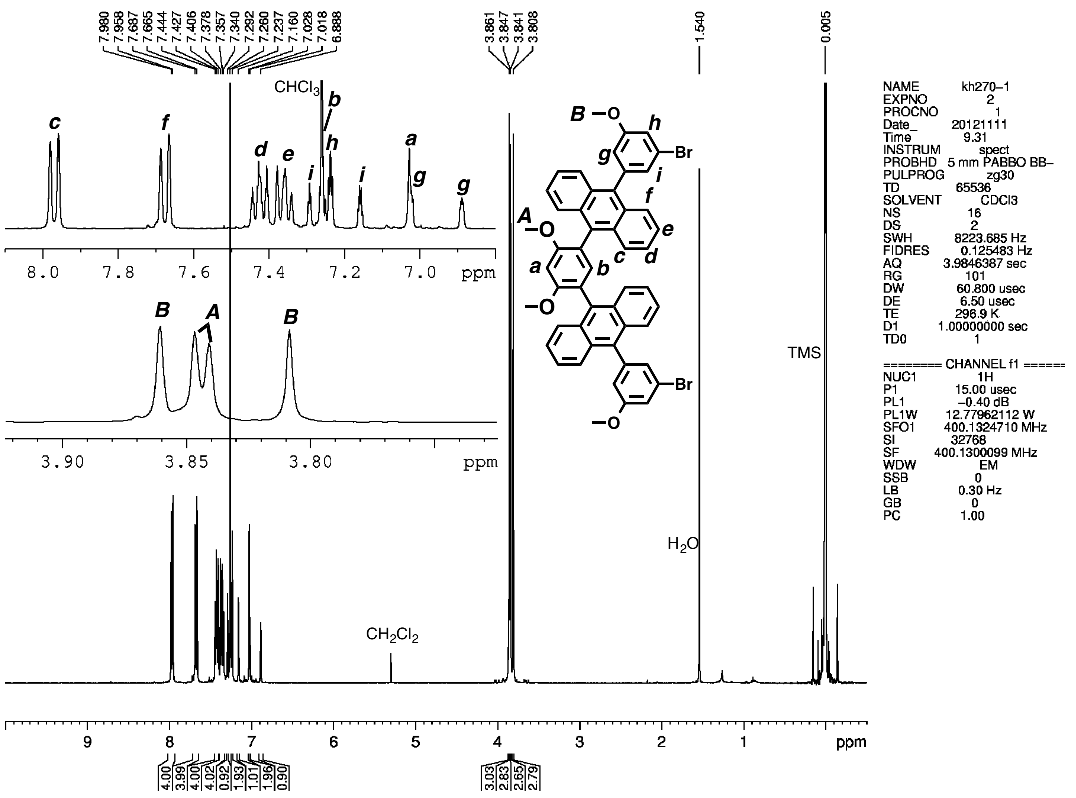

Figure S3. ^{1}H NMR spectrum (400 MHz, $CDCl_3$, r.t.) of **2a**.

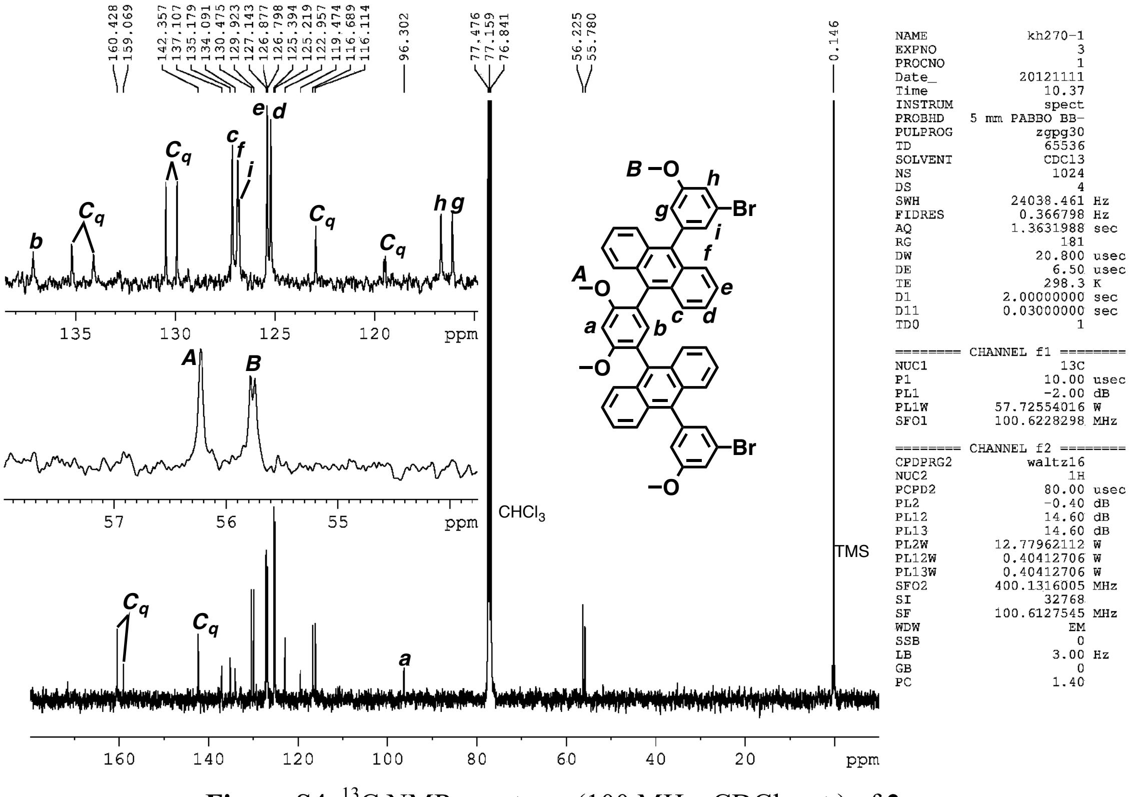

Figure S4. ^{13}C NMR spectrum (100 MHz, $CDCl_3$, r.t.) of **2a**.

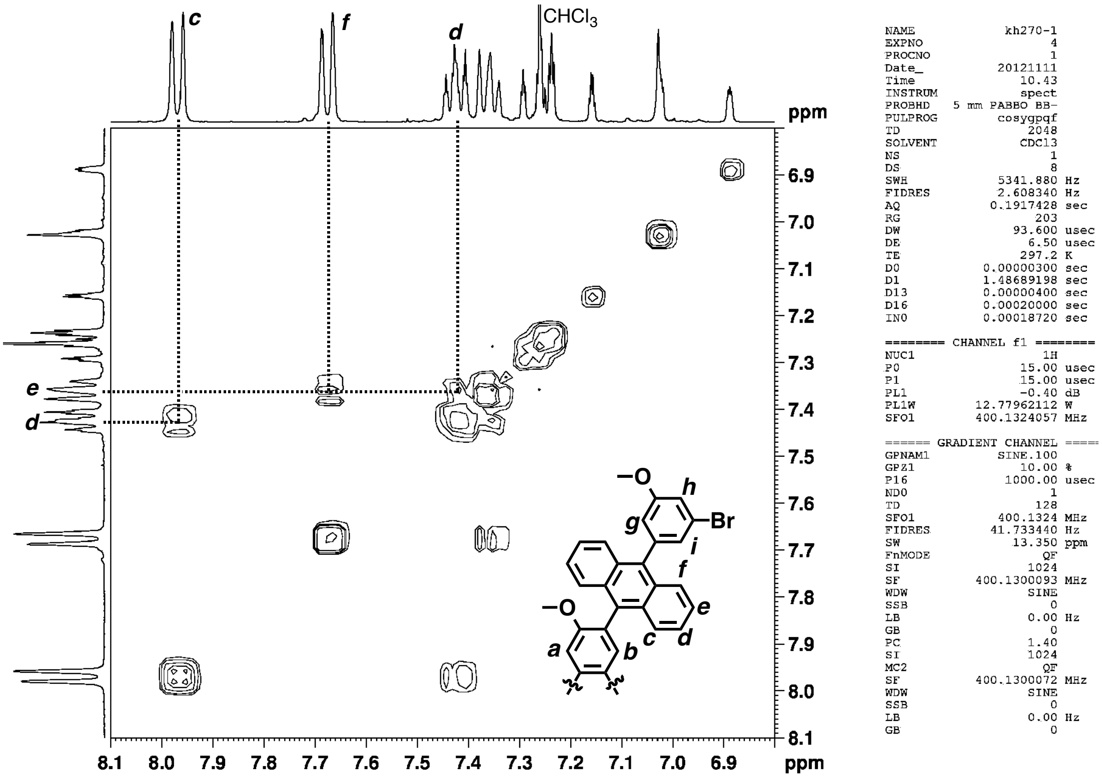

Figure S5. ^{1}H-^{1}H COSY spectrum (400 MHz, $CDCl_3$, r.t.) of **2a** (aromatic region).

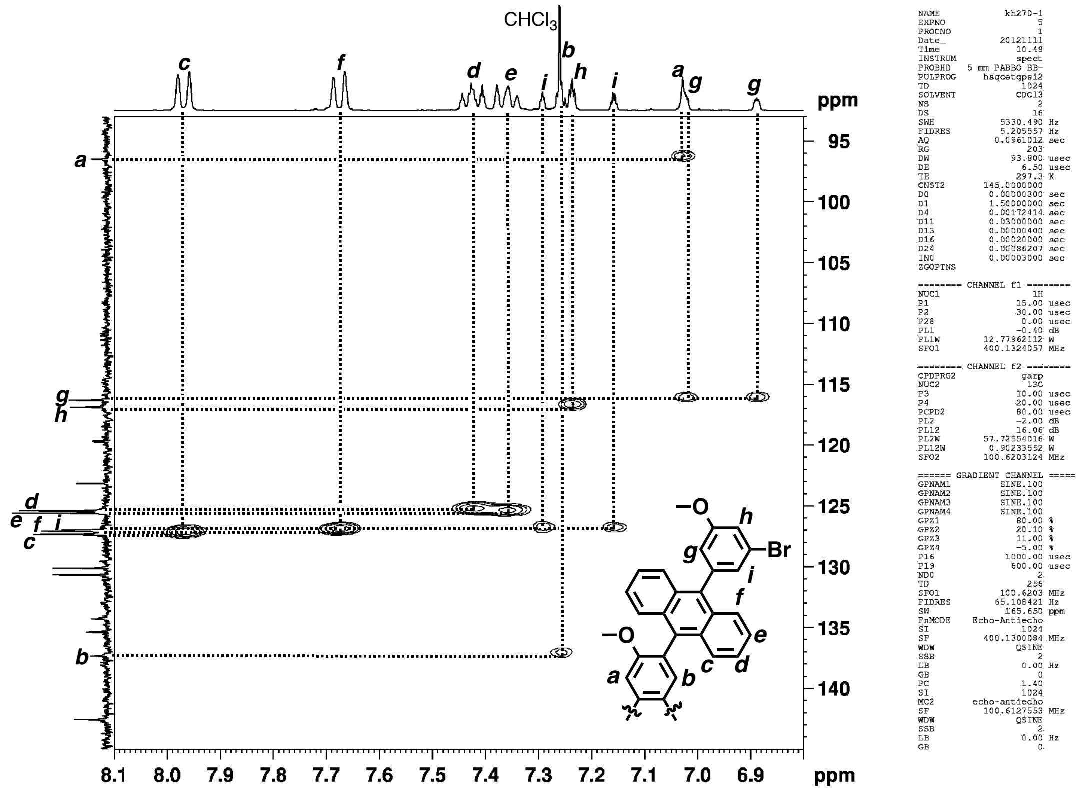

Figure S6. HSQC spectrum (400 MHz, $CDCl_3$, r.t.) of **2a** (aromatic region).

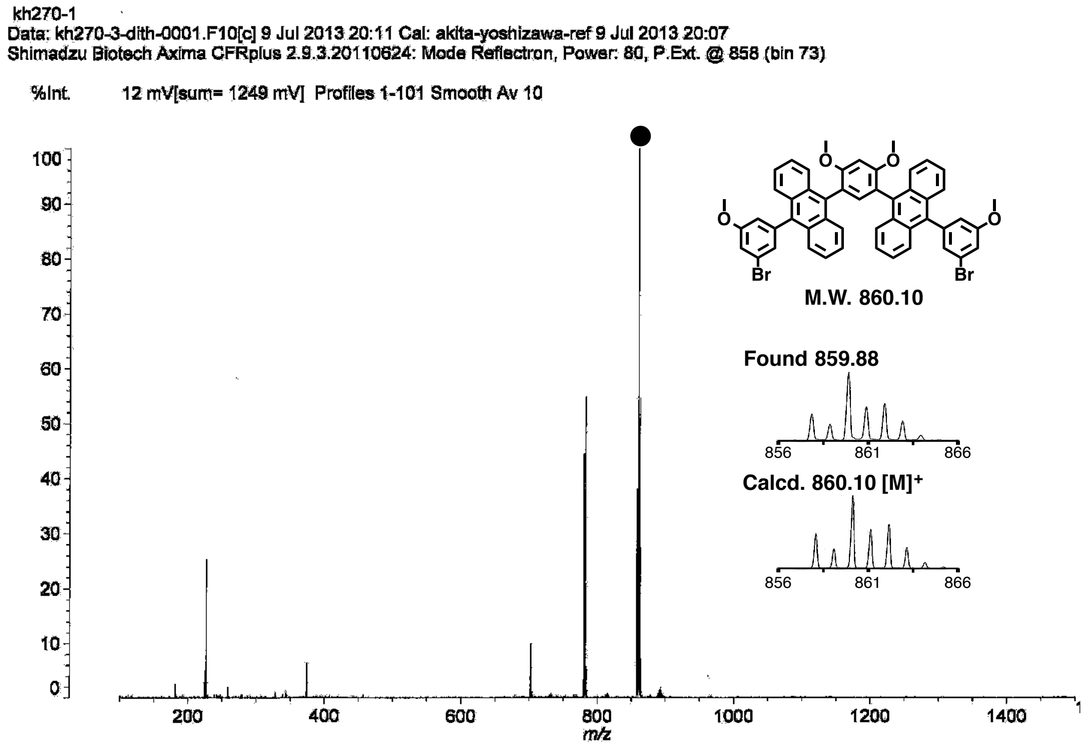

Figure S7. MALDI-TOF MS spectrum (dithranol) of **2a**.

Synthesis of half-tube 2b KH-279, (286, 298)

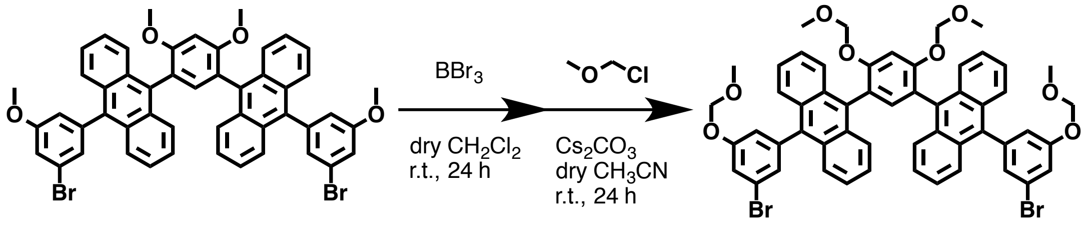

Dry CH_2Cl_2 (50 mL) and half-tube **2a** (0.500 g, 0.581 mmol) were added to a 2-necked 200 mL glass flask filled with N_2. A CH_2Cl_2 solution (1.0 M) of BBr_3 (5.4 mL, 5.4 mmol) was slowly added to the solution at 0 °C and then the combined solution was stirred at 40 °C for 12 h. The reaction was quenched with H_2O. The two layers were separated and the aqueous layer was extracted with CH_2Cl_2. The combined organic layers were dried over $MgSO_4$, filtrated, and concentrated under reduced pressure. The resultant solid was washed with H_2O and hexane to afford a yellow solid. The resulted solid, Cs_2CO_3 (1.21 g, 3.73 mmol), and dry CH_3CN (30 mL) were added to a 2-necked 300 mL glass flask filled with N_2. After the mixture was stirred at r.t. for 30 min,

chloromethyl methyl ether (0.45 g, 5.7 mmol) was added to the solution. The resultant solution was stirred at r.t. for 16 h. The reaction was quenched with H_2O. The crude product was extracted with CH_2Cl_2 and the combined organic phase was dried over $MgSO_4$, filtrated, and concentrated under reduce pressure. The crude product was purified by silica-gel column chromatography (hexane:ethyl acetate = 10:1) to give **2b** as a yellow solid (0.400 g, 0.408 mmol, 70%).

^{1}H NMR (400 MHz, $CDCl_3$, r.t.): δ 8.00 (d, J = 8.4 Hz, 4H), 7.69 (d, J = 8.4 Hz, 4H), 7.47-7.35 (m, 12H), 7.29 (s, 1H), 7.22 (s, 1H), 7.15 (s, 1H), 7.02 (s, 1H), 5.24 (s, 2H), 5.19 (s, 2H), 5.11 (s, 4H), 3.53 (s, 3H), 3.49 (s, 3H), 3.22 (s, 3H), 3.20 (s, 3H). ^{13}C NMR (100 MHz, $CDCl_3$, r.t.): δ 158.2 (C_q), 156.6 (C_q), 142.4 (C_q), 137.0 (CH), 135.1 (C_q), 134.1 (C_q), 130.5 (C_q), 129.9 (C_q), 128.1 (CH), 127.2 (CH), 126.8 (CH), 125.5 (CH), 125.3 (CH), 122.9 (C_q), 122.2 (C_q), 118.9 (CH), 118.8 (CH), 118.5 (CH), 103.4 (CH), 95.0 (CH_2), 56.4 (CH_3). FT-IR (KBr, cm^{-1}): 3438, 3068, 2949, 2916, 2839, 1597, 1566, 1371, 1248, 1151, 1072, 1018, 920, 766. MALDI-TOF MS (dithranol): m/z Calcd. for $C_{54}H_{44}Br_2O_8$ $[M]^+$ 980.14, Found 979.93. E.A.: Calcd. for $C_{54}H_{44}Br_2O_8$: C, 66.13; H, 4.52. Found: C, 66.13; H, 4.25.

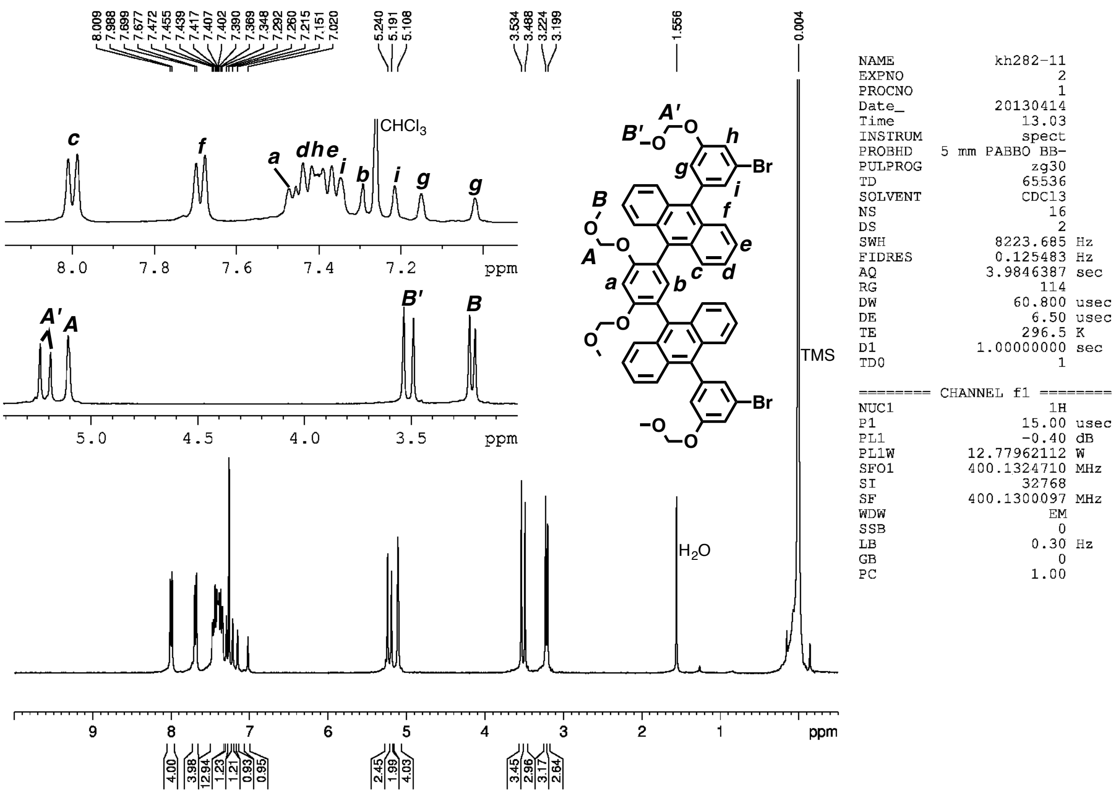

Figure S8. ^{1}H NMR spectrum (400 MHz, $CDCl_3$, r.t.) of **2b**.

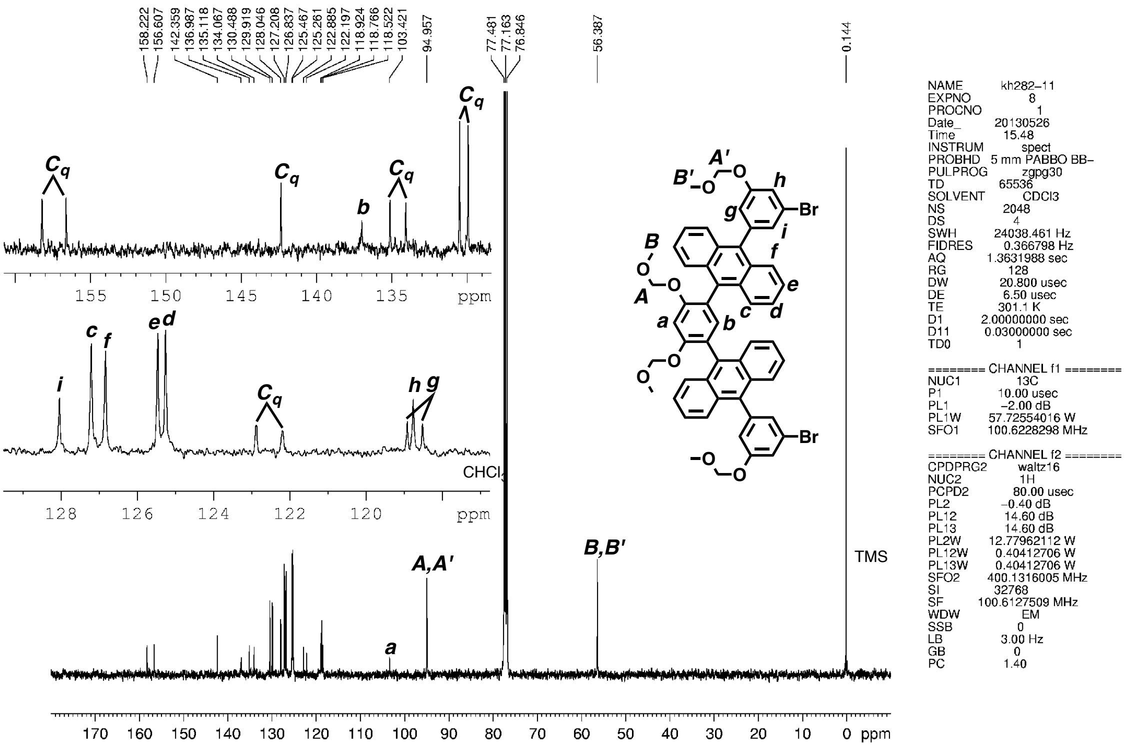

Figure S9. ^{13}C NMR spectrum (100 MHz, $CDCl_3$, r.t.) of **2b**.

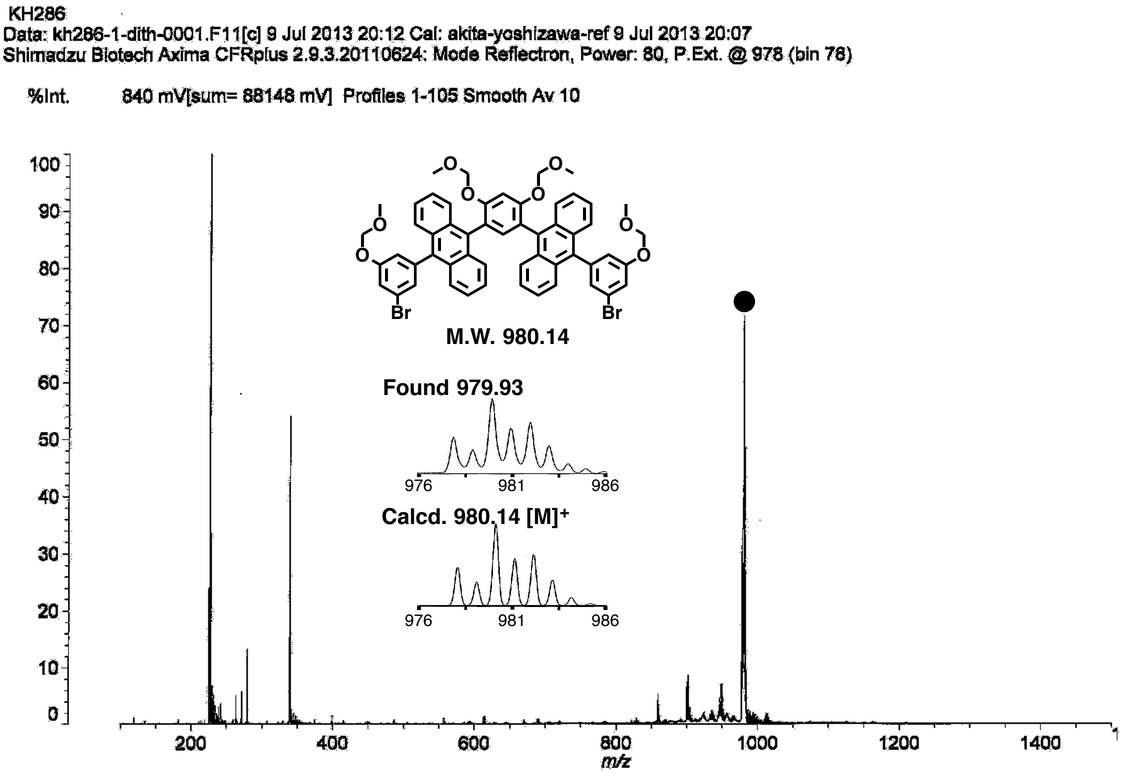

Figure S10. MALDI-TOF MS spectrum (dithranol) of **2b**.

Synthesis of tube 1” KH-304, (282, 289, 313)

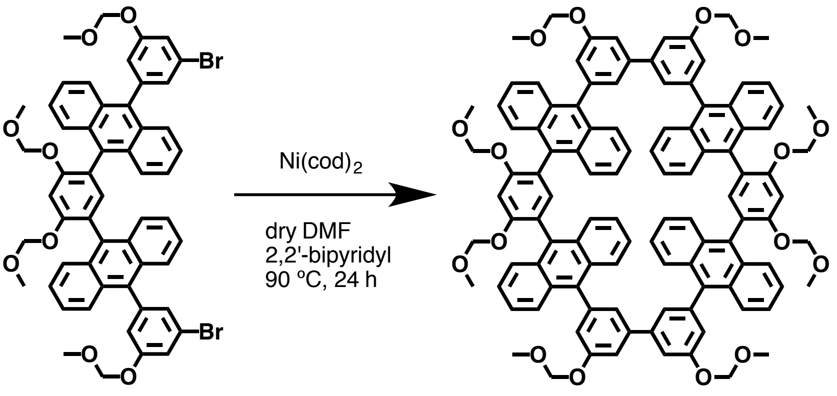

Half-tube **2b** (0.150 g, 0.153 mmol), Ni(cod)$_2$ (0.098 g, 0.36 mmol), 2,2’-bipyridyl (0.064 g, 0.41 mmol), and dry DMF (50 mL) were added to a 2-necked 100 mL glass flask filled with N_2 and the resultant mixture was stirred at 90 ºC for 24 h. The reaction was quenched with H_2O. The two layers were separated and the aqueous layer was extracted with CH_2Cl_2. The combined organic layers were concentrated under reduced pressure and washed with H_2O, CH_3OH, and acetone. The crude product was purified by silica-gel column chromatography (hexane:$CHCl_3$ = 10:1) to give **1”** as a yellow solid (0.037 g, 0.023 mmol, 30%).

^{1}H NMR (400 MHz, $CDCl_3$, r.t.): δ 7.82 (d, J = 8.4 Hz, 8H), 7.65 (m, 4H), 7.61 (d, J = 8.4 Hz, 4H), 7.40 (s, 2H), 7.27-7.18 (m, 24H), 7.03 (s, 2H), 5.34 (s, 8H), 5.04 (s, 8H), 3.60 (s, 12H), 3.19 (s, 12H). FT-IR (KBr, cm^{-1}): 3460, 3063, 2952, 2925, 2851, 2827, 1587, 1379, 1150, 1082, 1000, 923, 768. MALDI-TOF MS (dithranol): m/z Calcd. for $C_{108}H_{88}O_{16}$ [M]$^+$ 1641.61, Found 1641.44. HR MS (ESI): m/z Calcd. for $C_{108}H_{88}O_{16}$ [M]$^+$ 1641.6106, Found 1641.6082.

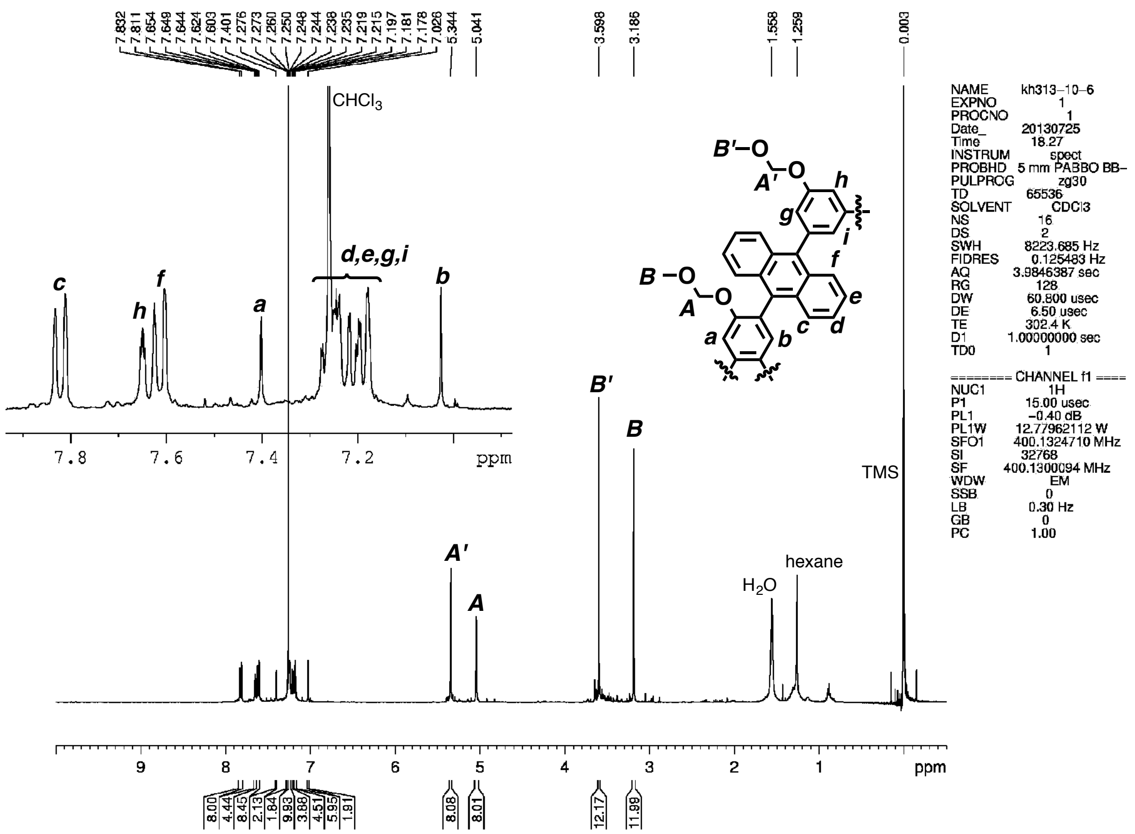

Figure S11. ^1H NMR spectrum (400 MHz, $CDCl_3$, r.t.) of **1”**.

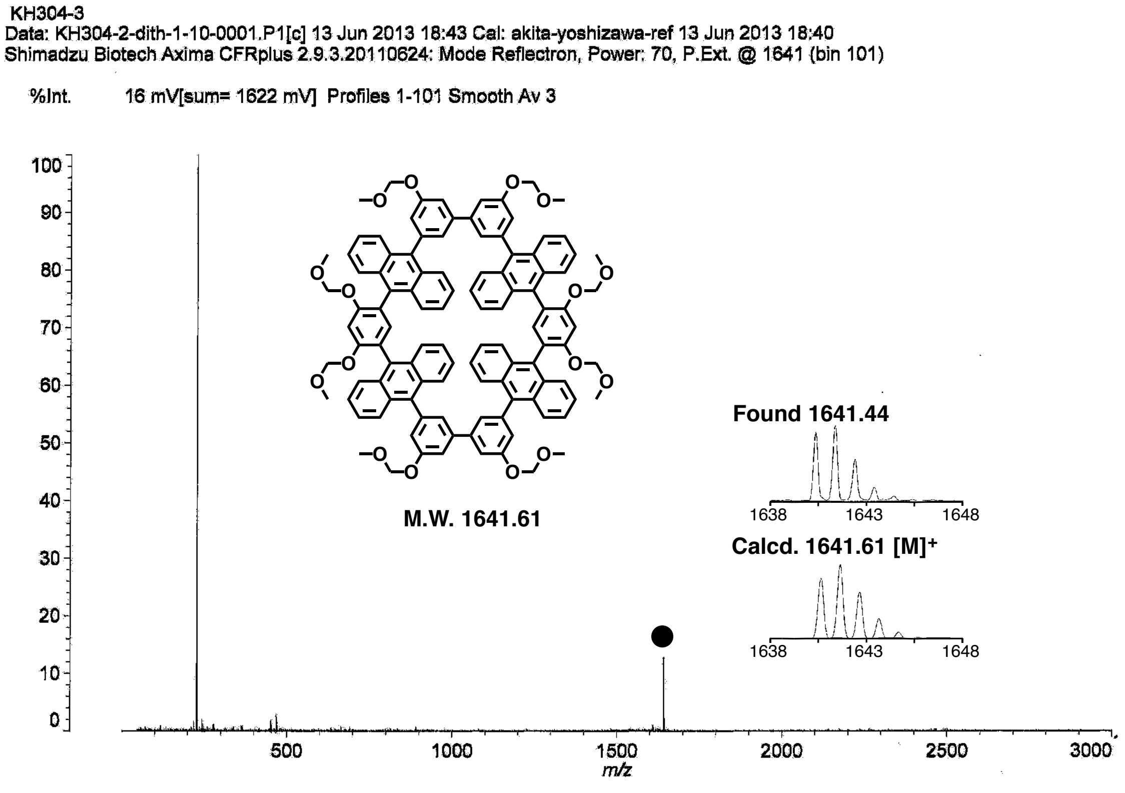

Figure S12. MALDI-TOF MS spectrum (dithranol) of **1”**.

Synthesis of tube 1 KH-349, (282, 295, 306, 322)

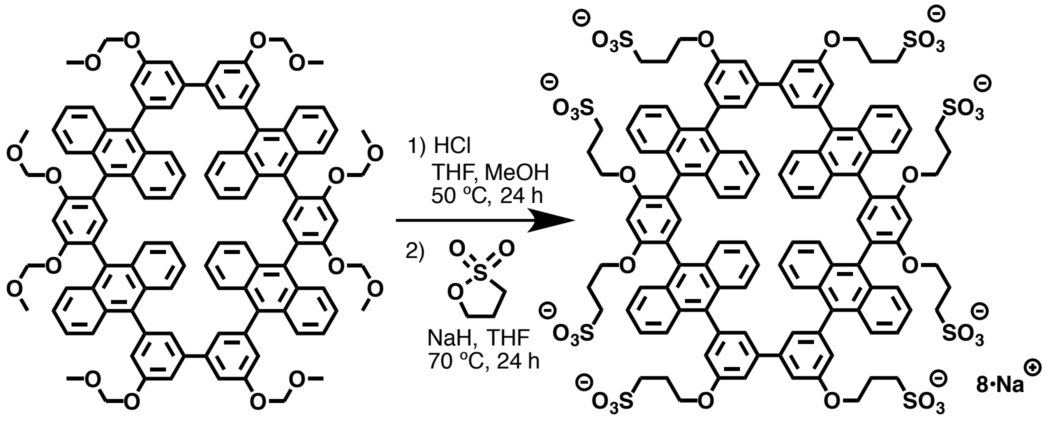

Tube **1”** (74.3 mg, 0.0452 mmol), THF (40 mL), and methanol (10 mL) were added to a 100 mL glass flask. Concentrated hydrochloric acid (50 mL) was added to this flask and stirred at 50 ºC for 24 h. The mixture was concentrated under reduce pressure. The crude product was washed with H_2O and $CHCl_3$, and purified by silica-gel column chromatography (hexane:acetone = 1:1) to give a deprotected tube as a white solid. NaH (60% in oil; 44.5 mg, 1.11 mmol) was added to a 100 mL glass flask and washed with hexane under N_2. The resultant deprotected tube and dry THF (20 mL) were added to this flask and stirred at r.t. for 1 h. 1,3-Propanesultone (0.135 g, 1.11 mmol) was added dropwise to this flask. The resultant mixture was stirred overnight at 80 ºC. The mixture was concentrated under reduce pressure and the crude product was washed with ether, acetone, and 1-propanol to afford **1** as a yellow solid (54.9 mg, 0.0225 mmol, 50%).

^{1}H NMR (400 MHz, CD_3OD, r.t.): δ 7.79 (d, J = 8.4 Hz, 8H), 7.65 (s, 4H), 7.62 (d, J = 8.4 Hz, 4H), 7.31-7.22 (m, 18H), 7.12 (s, 4H), 7.09 (m, 4H), 6.83 (s, 2H), 4.33 (t, J = 6.0 Hz, 8H), 4.23 (t, J = 6.0 Hz, 8H), 3.12 (t, J = 7.6 Hz, 8H), 2.47 (t, J = 7.6 Hz, 8H), 2.37 (q, J = 7.2 Hz, 8H), 1.95 (q, J = 7.2 Hz, 8H). ^{13}C NMR (100 MHz, CD_3OD, r.t.): δ 161.2 (C_q), 159.4 (C_q), 142.7 (C_q), 142.5 (C_q), 137.9 (CH), 137.5 (C_q), 134.9 (C_q), 131.5 (C_q), 131.0 (C_q), 127.9 (CH), 127.8 (CH), 126.1 (CH), 125.9 (CH), 123.0 (CH), 121.1 (C_q), 118.1 (CH), 113.4 (CH), 99.9 (CH), 68.6 (CH_2), 68.2 (CH_2), 49.4-48.6 (overlapped with MeOH), 26.5 (CH_2), 26.1 (CH_2). FT-IR (KBr, cm^{-1}): 3451, 1504, 1440, 1380, 1311, 1190, 1101, 1046, 801, 770, 606, 528. ESI-TOF MS (CH_3OH): m/z 383.8 [**1** – 6Na$^+$]$^{6-}$, 465.2 [**1** – 5Na$^+$]$^{5-}$, 587.2 [**1** – 4Na$^+$]$^{4-}$, 791.0 [**1** – 3Na$^+$]$^{3-}$.

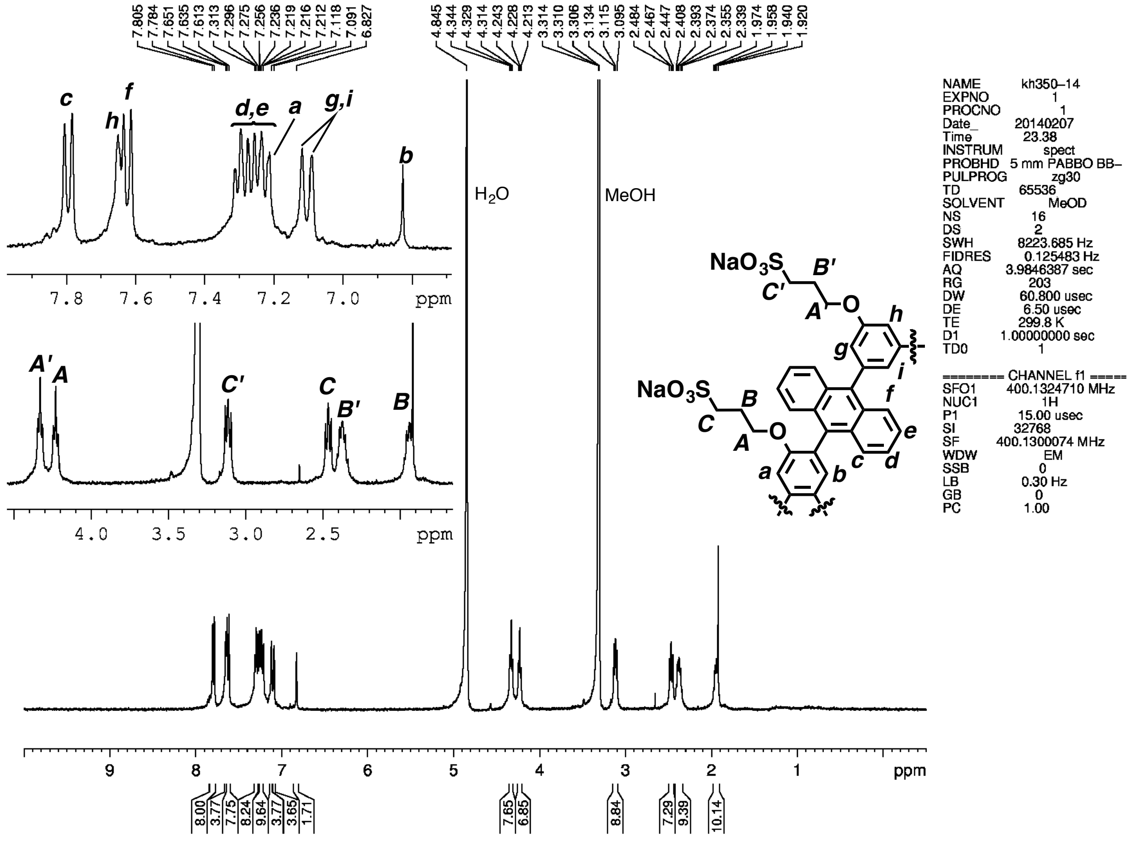

Figure S13. ^{1}H NMR spectrum (400 MHz, CD_3OD, r.t.) of **1**.

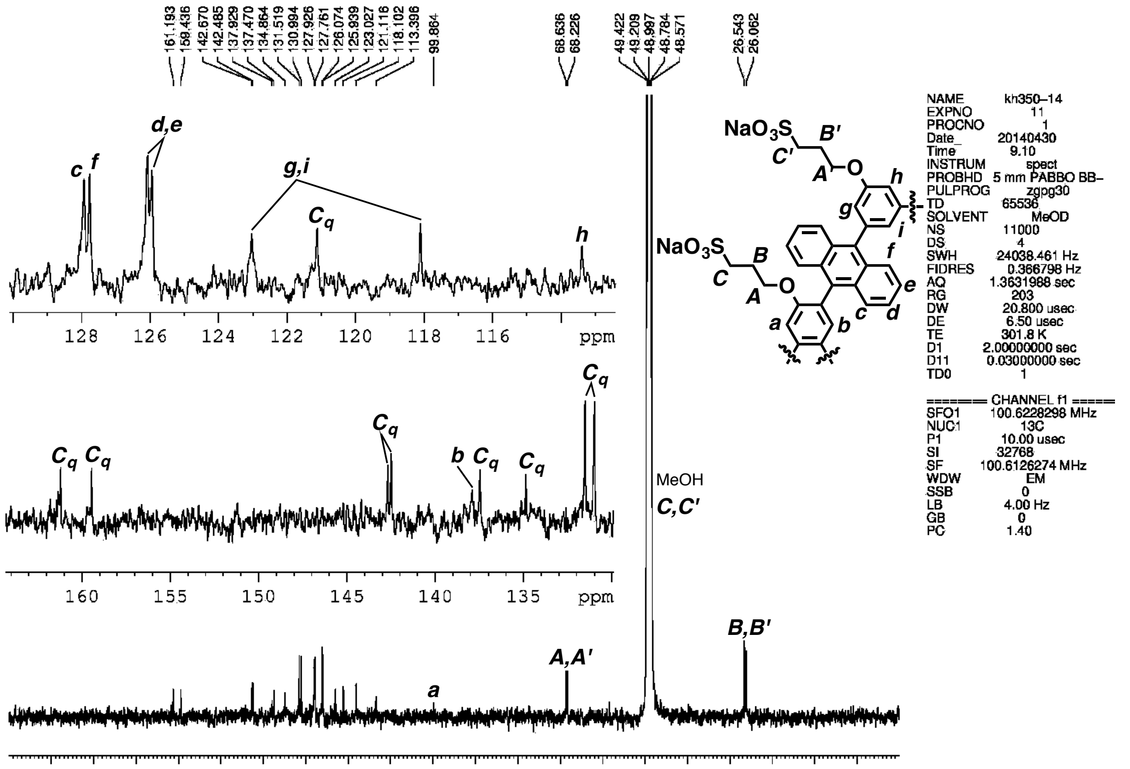

Figure S14. ^{13}C NMR spectrum (100 MHz, CD_3OD, r.t.) of **1**.

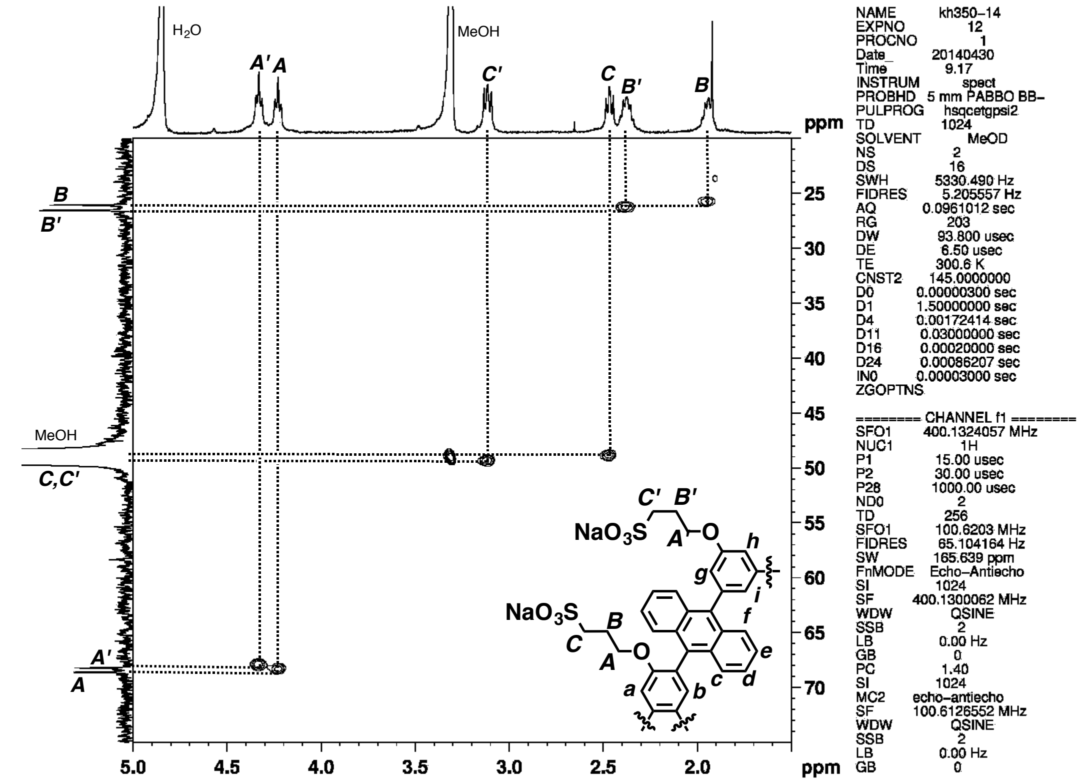

Figure S15. HSQC spectrum (400 MHz, CD_3OD, r.t.) of **1** (aliphatic region).

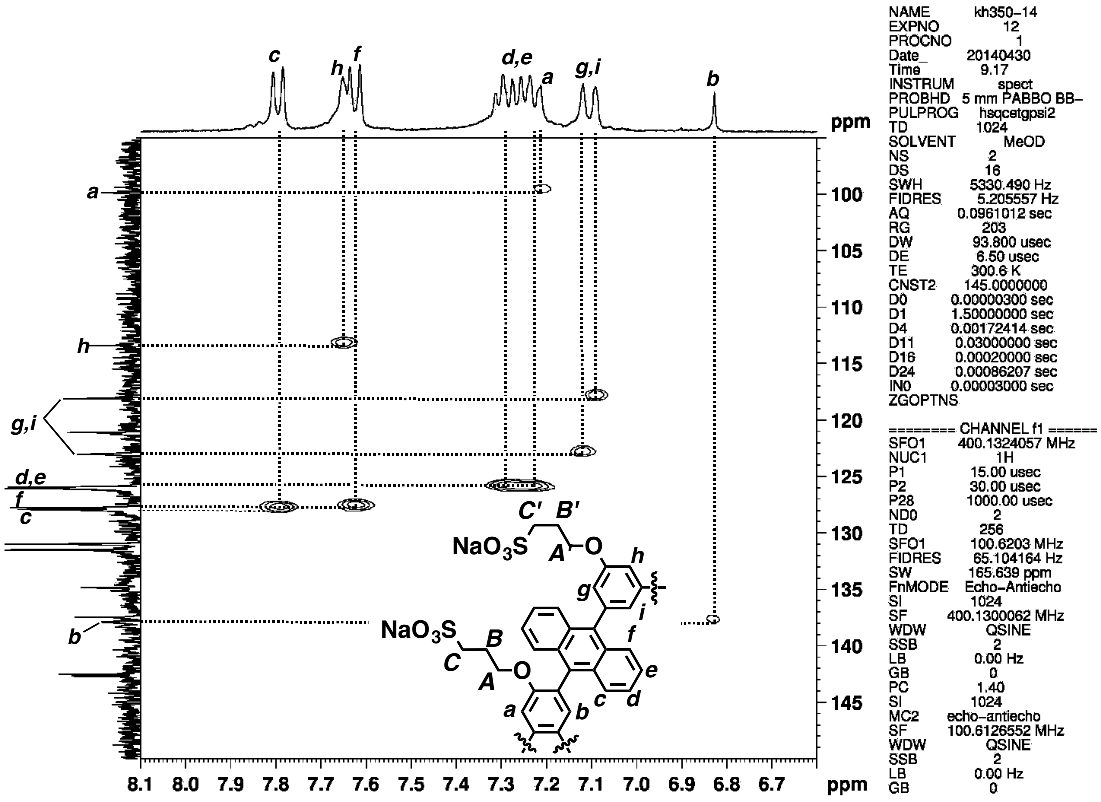

Figure S16. HSQC spectrum (400 MHz, CD_3OD, r.t.) of **1** (aromatic region).

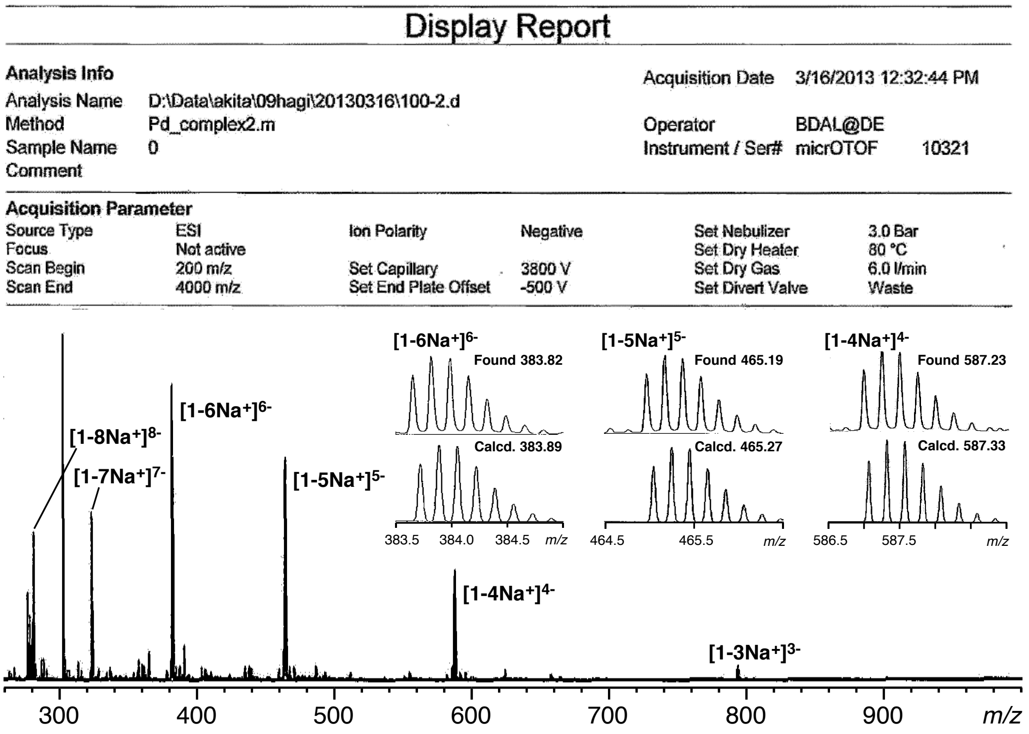

Figure S17. ESI-TOF MS spectrum (CH_3OH) of **1**.

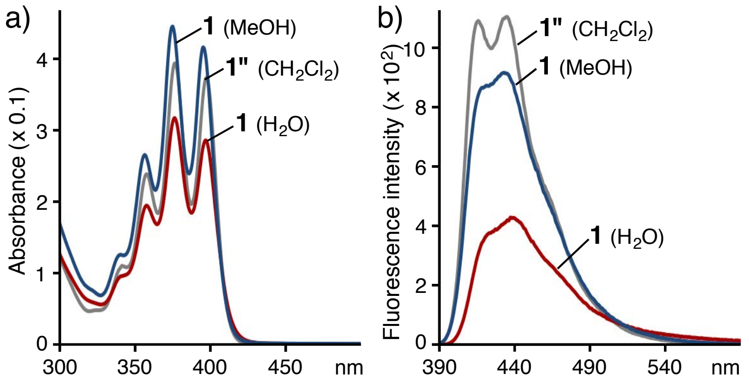

Figure S18. (a) UV-vis (10 μM, r.t.) and (b) fluorescence spectra (λ_{ex} = 377 nm, 10 μM, r.t.) of **1”** in CH_2Cl_2 and **1** in H_2O and CH_3OH.

Synthesis and properties of 1⊃(4a)$_2$ KH-347

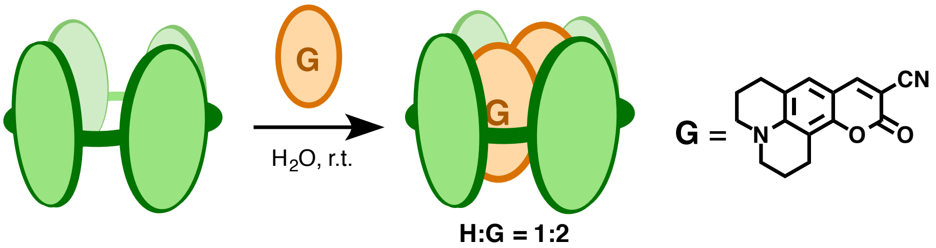

Coumarin 337 (**4a**; 0.05 mg, 0.2 μmol) was added to an H_2O solution (0.5 mL) of tube **1** (0.25 mg, 0.10 μmol) in a glass test tube. The solution was stirred at r.t. for 1 h. After filtration, the quantitative formation of a **1**⊃(**4a**)$_2$ complex was confirmed by UV-vis, fluorescence, DLS, and MS analyses.

ESI-TOF MS (H_2O): *m/z* 348.8 [**1**⊃(**4a**)$_2$ – 8Na$^+$]$^{8-}$, 401.9 [**1**⊃(**4a**)$_2$ – 7Na$^+$]$^{7-}$, 472.7 [**1**⊃(**4a**)$_2$ – 6Na$^+$]$^{6-}$, 571.7 [**1**⊃(**4a**)$_2$ – 5Na$^+$]$^{5-}$, 720.6 [**1**⊃(**4a**)$_2$ – 4Na$^+$]$^{4-}$.

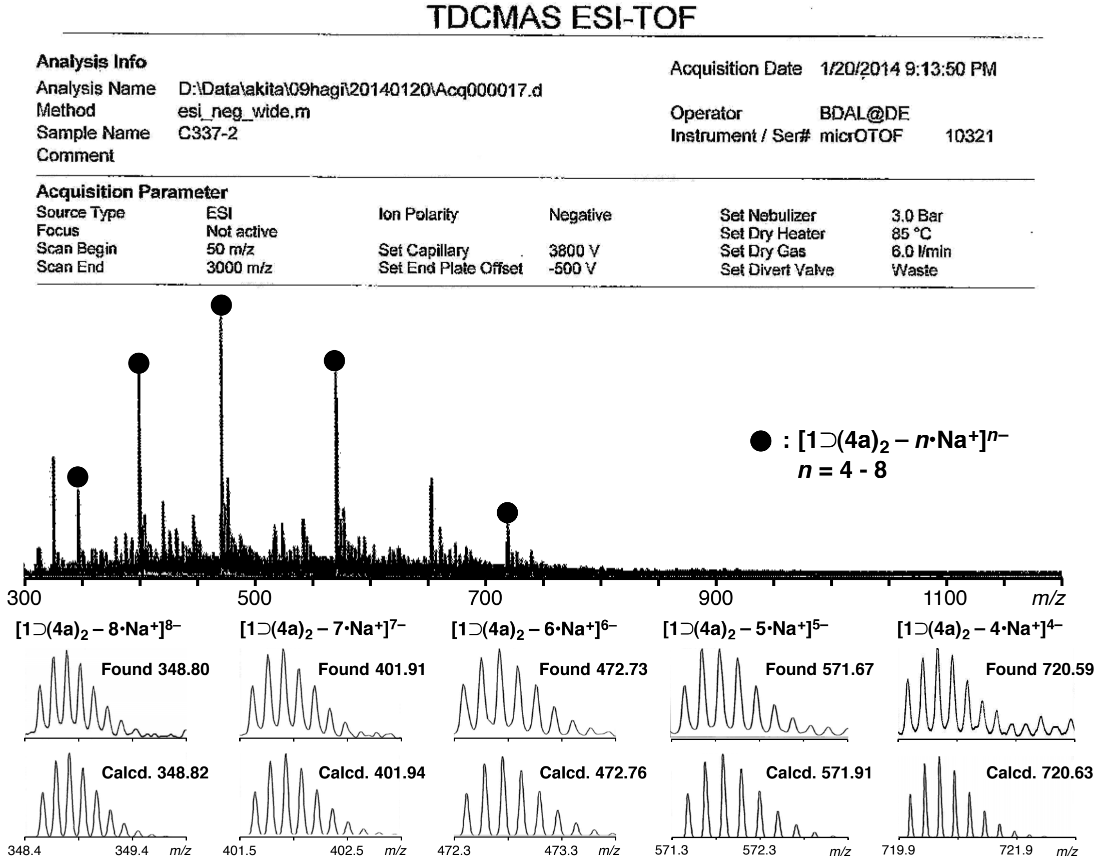

Figure S19. ESI-TOF MS spectrum (H_2O) of **1**⊃(**4a**)$_2$.

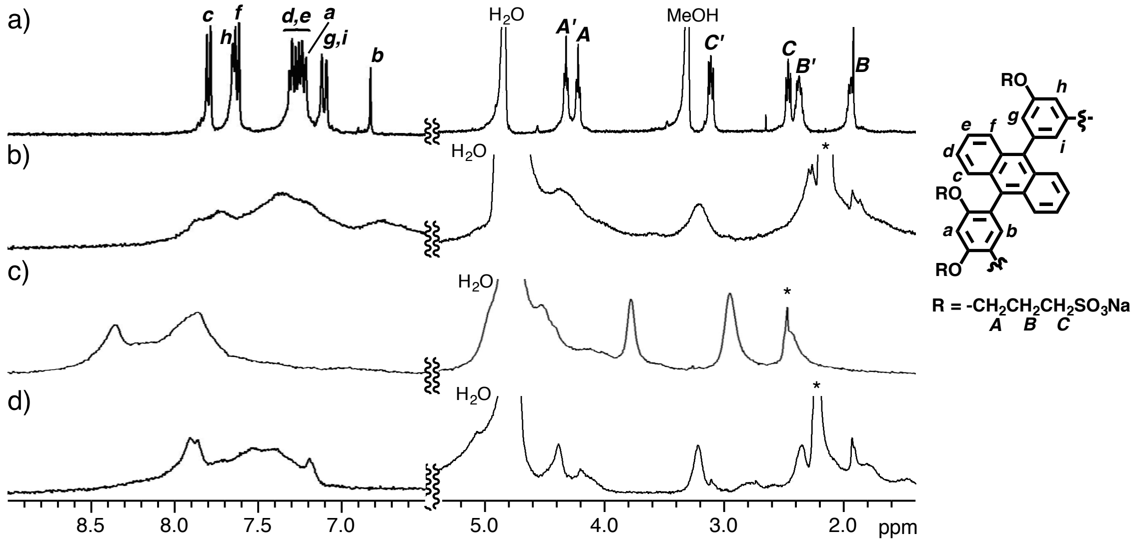

Figure S20. ^{1}H NMR spectra (400 MHz, r.t.) of tube **1** in (a) CD_3OD, (b) D_2O and (c) D_2O (at 80 ºC), and (d) **1**$\supset$(**4a**)$_2$ in D_2O.

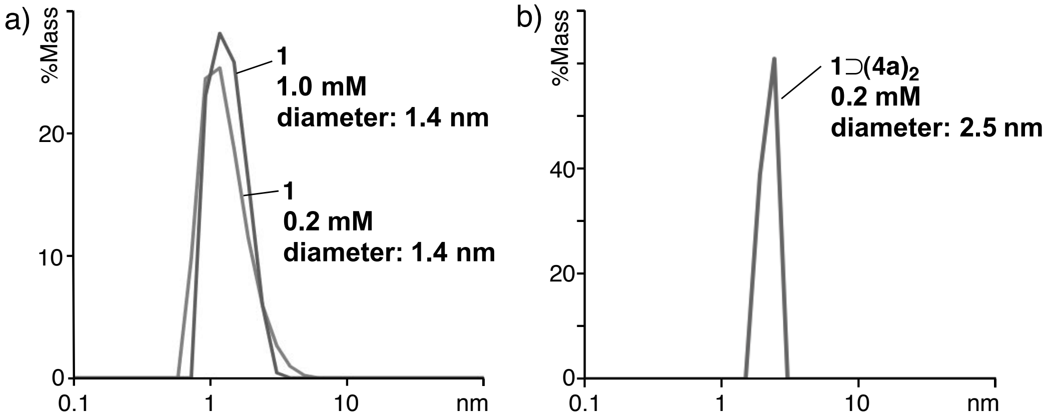

Figure S21. Particle size distribution (H_2O, r.t.) of (a) **1** and (b) **1**$\supset$(**4a**)$_2$ by DLS analysis.

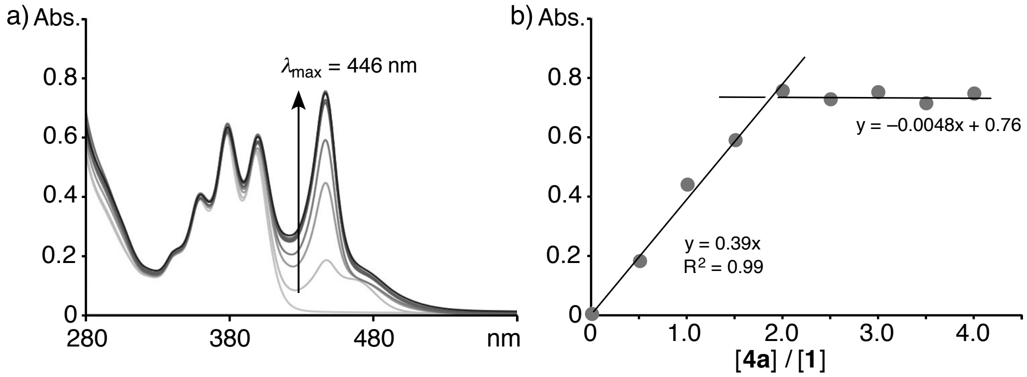

Figure S22. (a) Titration UV-vis spectra (0.2 mM, H_2O, r.t.) and (b) the plot (λ_{abs} = 446 nm) of **1** by the addition of **4a** ([**4a**]/[**1**] = 0.5, 1.0, 1.5, 2.0, 2.5, 3.0, 3.5, 4.0).

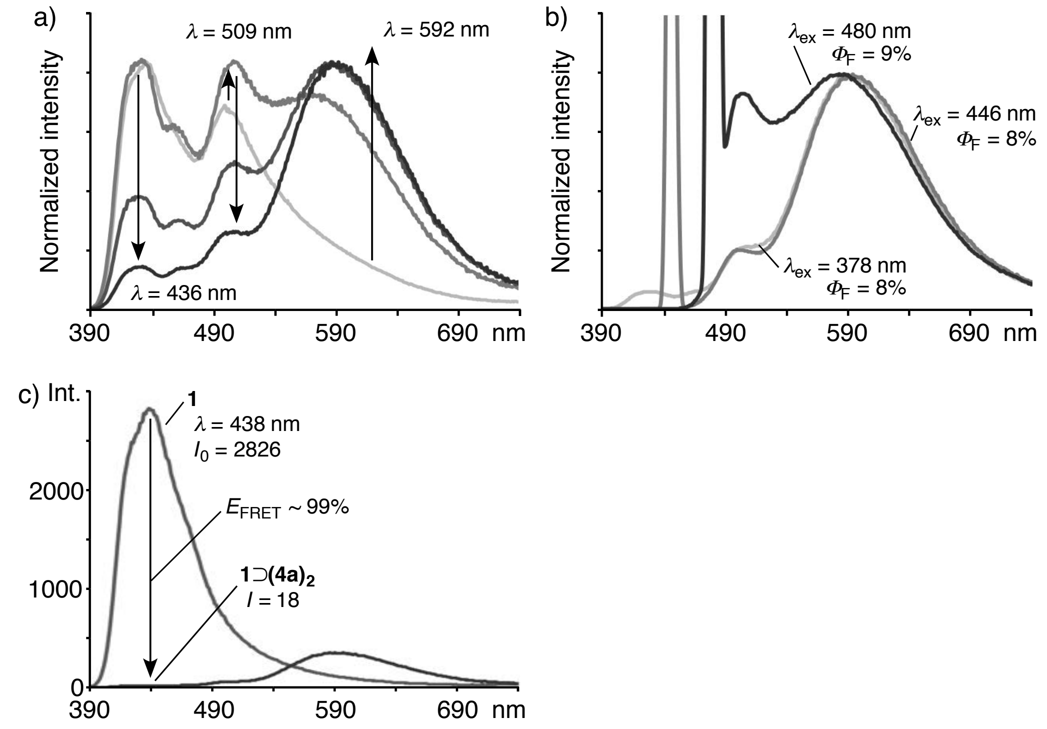

Figure S23. (a) Titration fluorescence spectra (λ_{ex} = 378 nm, 0.2 mM, H_2O, r.t.) of **1** by the addition of **4a ([4a]/[1]** = 0.5, 1.0, 1.5, 2.0), and (b) fluorescence spectra (0.2 mM, H_2O, r.t.) of **1**⊃(**4a**)$_2$ upon irradiation at λ_{ex} = 378, 446, and 480 nm, and (c) fluorescence spectra (0.2 mM, H_2O, r.t.) of **1** and **1**⊃(**4a**)$_2$ for the estimation of the FRET efficiency ($E_{FRET} = 1 - I/I_0$).

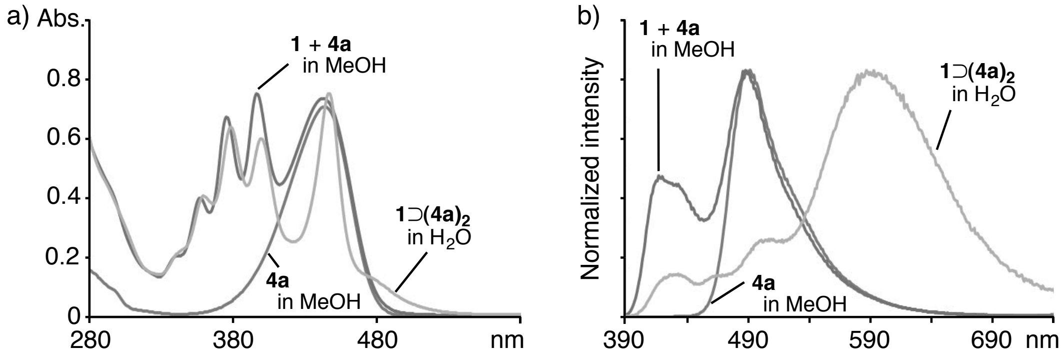

Figure S24. (a) UV-vis spectra (r.t.) and (b) fluorescence spectra (λ_{ex} = 378 nm r.t.) of **1⊃(4a)$_2$** in H_2O (0.2 mM), **1** + **4a** in CH_3OH (0.2 mM), and **4a** in CH_3OH (0.4 mM).

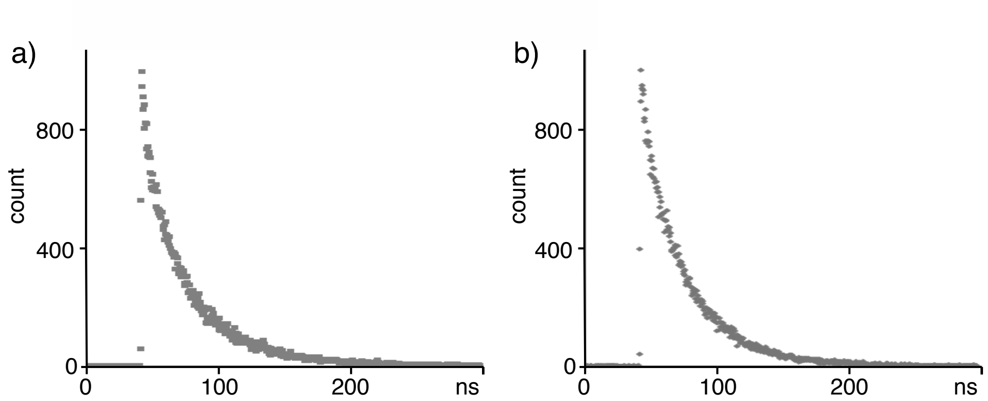

Figure S25. Fluorescent lifetime (λ_{ex} = 365 nm, 10 μM, H_2O, r.t.) of (a) **1** (λ_{em} = 440 nm) and (b) **1**⊃(**4a**)$_2$ (λ_{em} = 600 nm).

Table S1. Fluorescent lifetime of **1** and **1**⊃(**4a**)$_2$.

	τ_1 [ns]	τ_2 [ns]	τ_3 [ns]	A_1	A_2	A_3	$<\tau>^a$ [ns]
**1**	0.443	33.9		618	329		12.1
**1**⊃(**4a**)$_2$	0.288	2.61	32.4	267	82.7	357	16.7

[a] $<\tau> = (A_1\tau_1 + A_2\tau_2 + A_3\tau_3)/(A_1 + A_2 + A_3)$

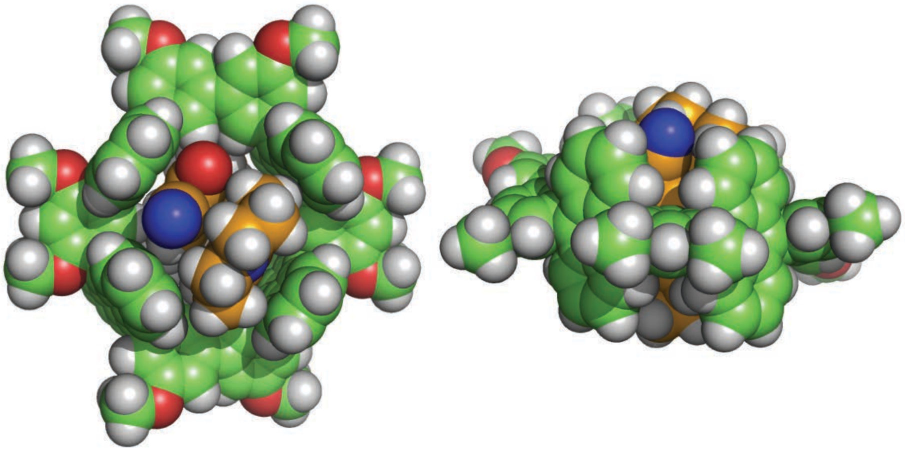

Figure S26. Optimized structure of a **1**⊃(**4a**)$_2$ complex.

Synthesis and properties of $1\supset(4b)_2$ KH-347

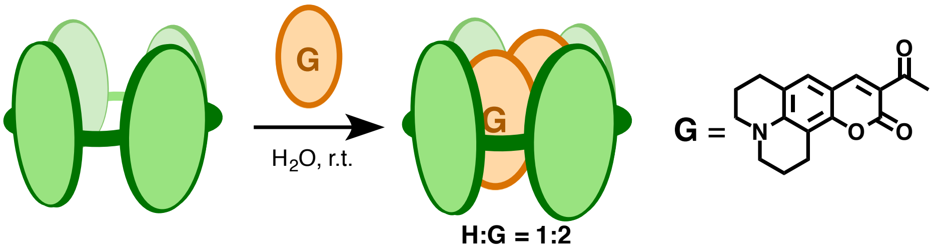

Coumarin 334 (**4b**; 0.06 mg, 0.2 μmol) was added to an H_2O solution (0.5 mL) of tube **1** (0.25 mg, 0.10 μmol) in a glass test tube. The solution was stirred at r.t. for 1 h. After filtration, the quantitative formation of a **1**⊃(**4b**)$_2$ complex was confirmed by UV-vis, fluorescence, and ESI-TOF MS analyses.

ESI-TOF MS (H_2O): *m/z* 406.9 [**1**⊃(**4b**)$_2$ – 7Na$^+$]$^{7-}$, 478.4 [**1**⊃(**4b**)$_2$ – 6Na$^+$]$^{6-}$, 578.7 [**1**⊃(**4b**)$_2$ – 5Na$^+$]$^{5-}$, 792.1 [**1**⊃(**4b**)$_2$ – 4Na$^+$]$^{4-}$, 979.8 [**1**⊃(**4b**)$_2$ – 3Na$^+$]$^{3-}$.

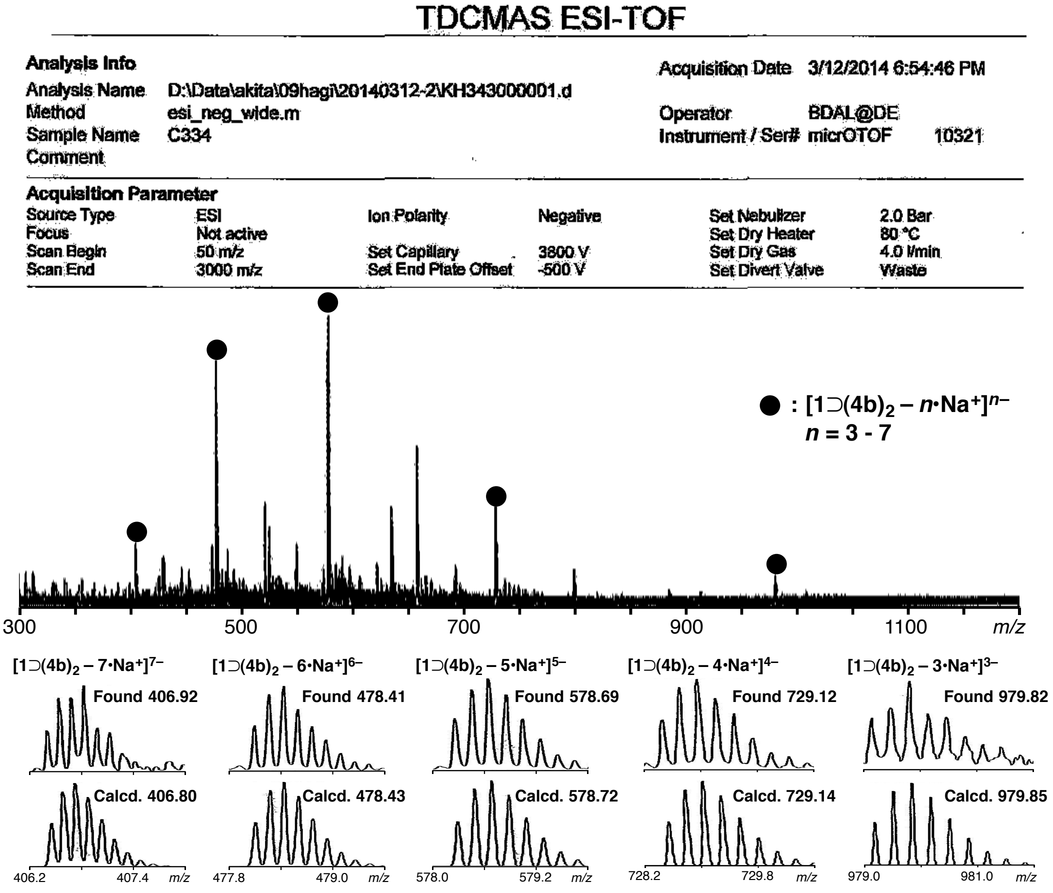

Figure S27. ESI-TOF MS spectrum (H_2O) of **1**⊃(**4b**)$_2$.

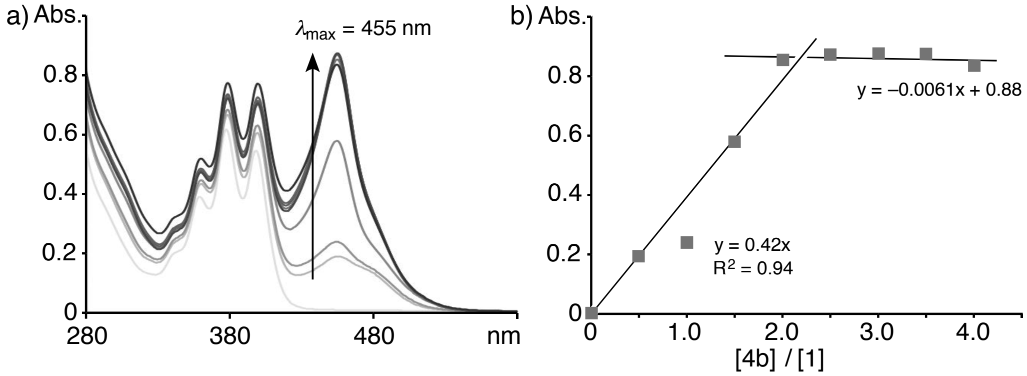

Figure S28. (a) Titration UV-vis spectra (0.2 mM, H_2O, r.t.) and (b) the plot (λ_{abs} = 455 nm) of **1** by the addition of **4b** ([**4b**]/[**1**] = 0.5, 1.0, 1.5, 2.0, 2.5, 3.0, 3.5, 4.0).

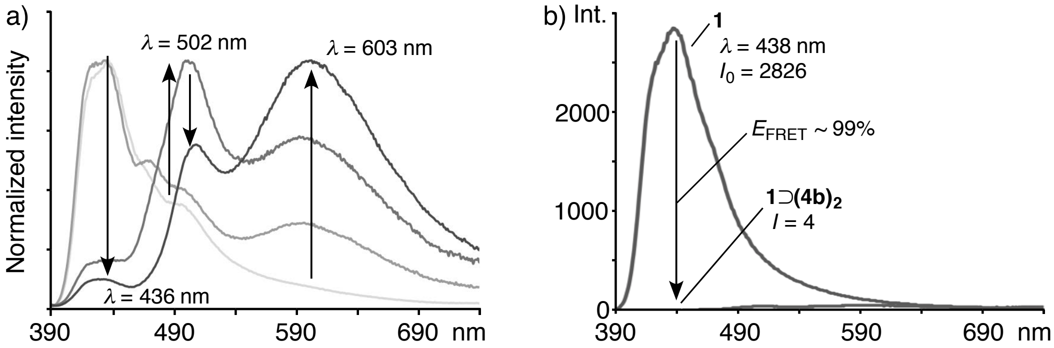

Figure S29. (a) Titration fluorescence spectra (λ_{ex} = 378 nm, 0.2 mM, H_2O, r.t.) of **1** by the addition of **4b** ([**4b**]/[**1**] = 0.5, 1.0, 1.5, 2.0), and (b) fluorescence spectra (0.2 mM, H_2O, r.t.) of **1** and **1**⊃(**4b**)$_2$ for the estimation of the FRET efficiency ($E_{FRET} = 1 - I/I_0$).

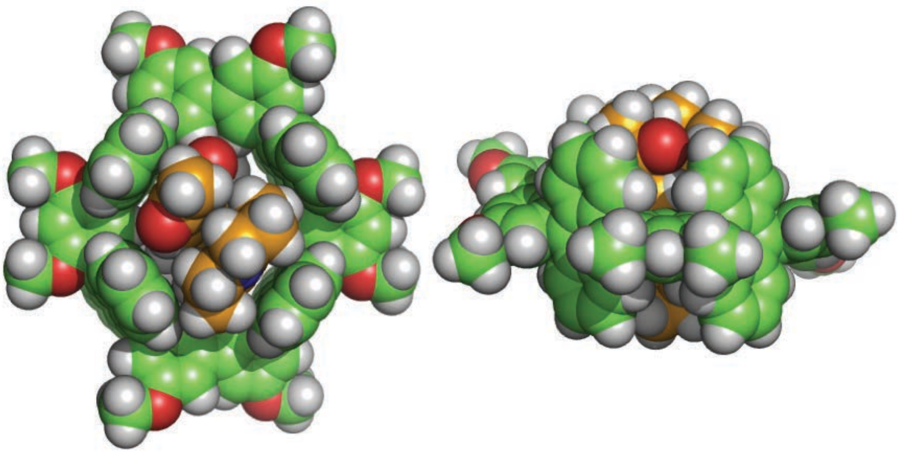

Figure S30. Optimized structure of a **1**⊃(**4b**)$_2$ complex.

Synthesis and properties of 1⊃4c KH-348

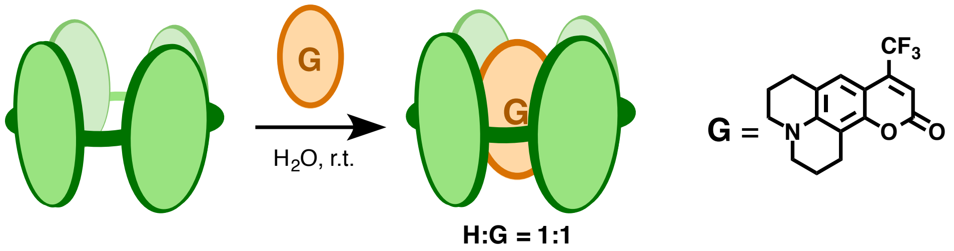

Coumarin 153 (**4c**; 0.06 mg, 0.2 μmol) was added to an H_2O solution (0.5 mL) of tube **1** (0.25 mg, 0.10 μmol) in a glass test tube. The solution was stirred at r.t. for 1 h. After filtration, the formation of a **1⊃4c** complex (40%) was confirmed by UV-vis, fluorescence, and ESI-TOF MS analyses.

ESI-TOF MS (H_2O): *m/z* 320.9 [**1⊃4c** – 8Na$^+$]$^{8-}$, 370.1 [**1⊃4c** – 7Na$^+$]$^{7-}$, 435.6 [**1⊃4c** – 6Na$^+$]$^{6-}$, 527.3 [**1⊃4c** – 5Na$^+$]$^{5-}$, 664.6 [**1⊃4c** – 4Na$^+$]$^{4-}$.

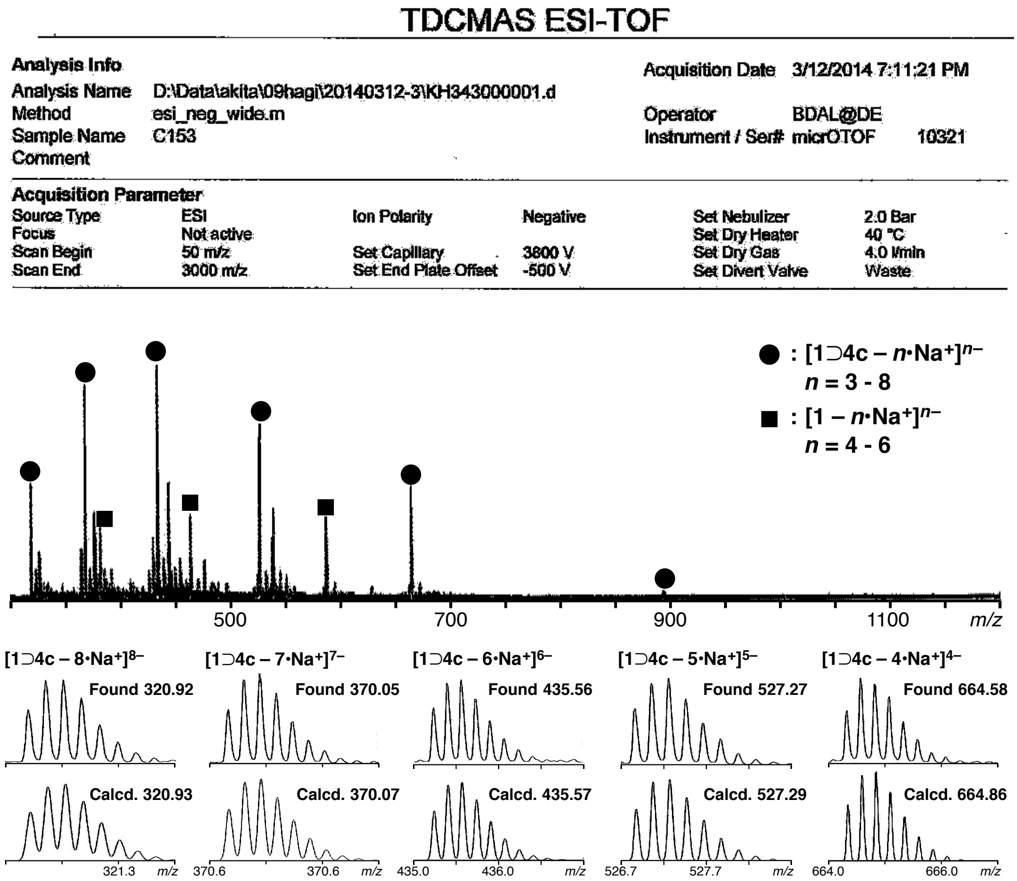

Figure S31. ESI-TOF MS spectrum (H_2O) of **1⊃4c**.

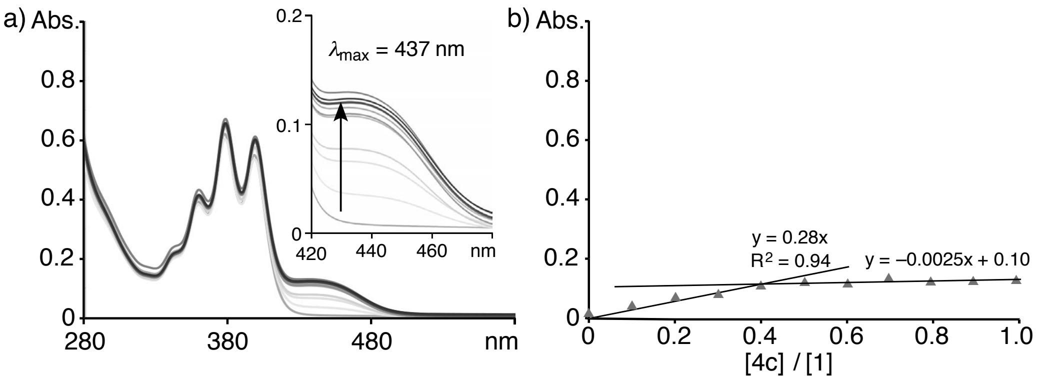

Figure S32. (a) Titration UV-vis spectra (0.2 mM, H_2O, r.t.) and (b) the plot (λ_{abs} = 437 nm) of **1** by the addition of **4c** ([**4c**]/[**1**] = 0.1, 0.2, 0.3, 0.4, 0.5, 0.6, 0.7, 0.8, 0.9, 1.0).

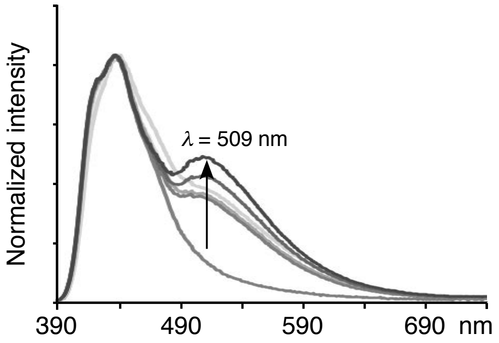

Figure S33. Titration fluorescence spectra (λ_{ex} = 378 nm, 0.2 mM, H_2O, r.t.) of **1** by the addition of **4c** ([**4c**]/[**1**] = 0.1, 0.2, 0.3, 0.4, 0.5).

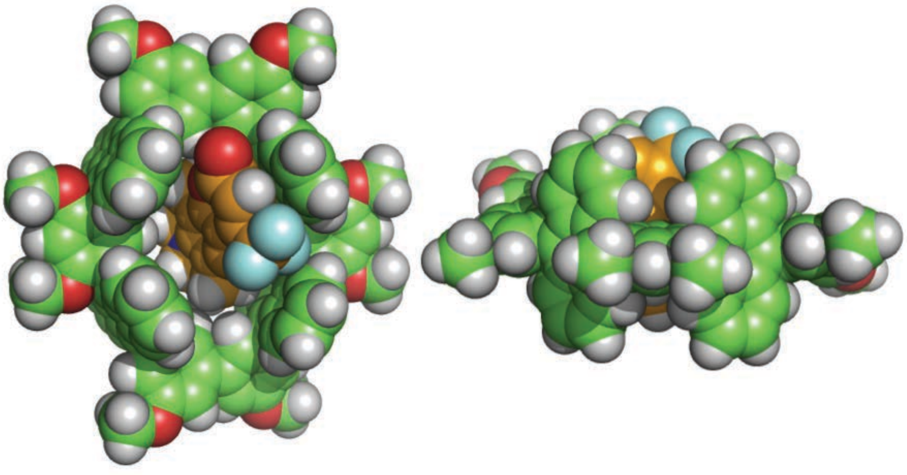

Figure S34. Optimized structure of **1**⊃**4c** complex.

Synthesis and properties of host-guest complex 1⊃4d   KH-348

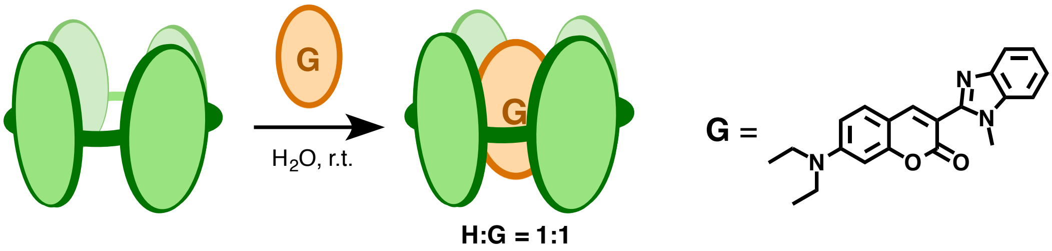

Coumarin 334 (**4d**; 0.07 mg, 0.2 μmol) was added to an H_2O solution (0.5 mL) of tube **1** (0.25 mg, 0.10 μmol) in a glass test tube. The solution was stirred at r.t. for 1 h. After filtration, the formation of a **1⊃4d** complex (30%) was confirmed by UV-vis, fluorescence, and ESI-TOF MS analyses.

ESI-TOF MS (H_2O): *m/z* 375.5 [**1⊃4d** – 7Na$^+$]$^{7–}$, 441.9 [**1⊃4d** – 6Na$^+$]$^{6–}$, 534.7 [**1⊃4d** – 5Na$^+$]$^{5–}$, 674.3 [**1⊃4d** – 4Na$^+$]$^{4–}$.

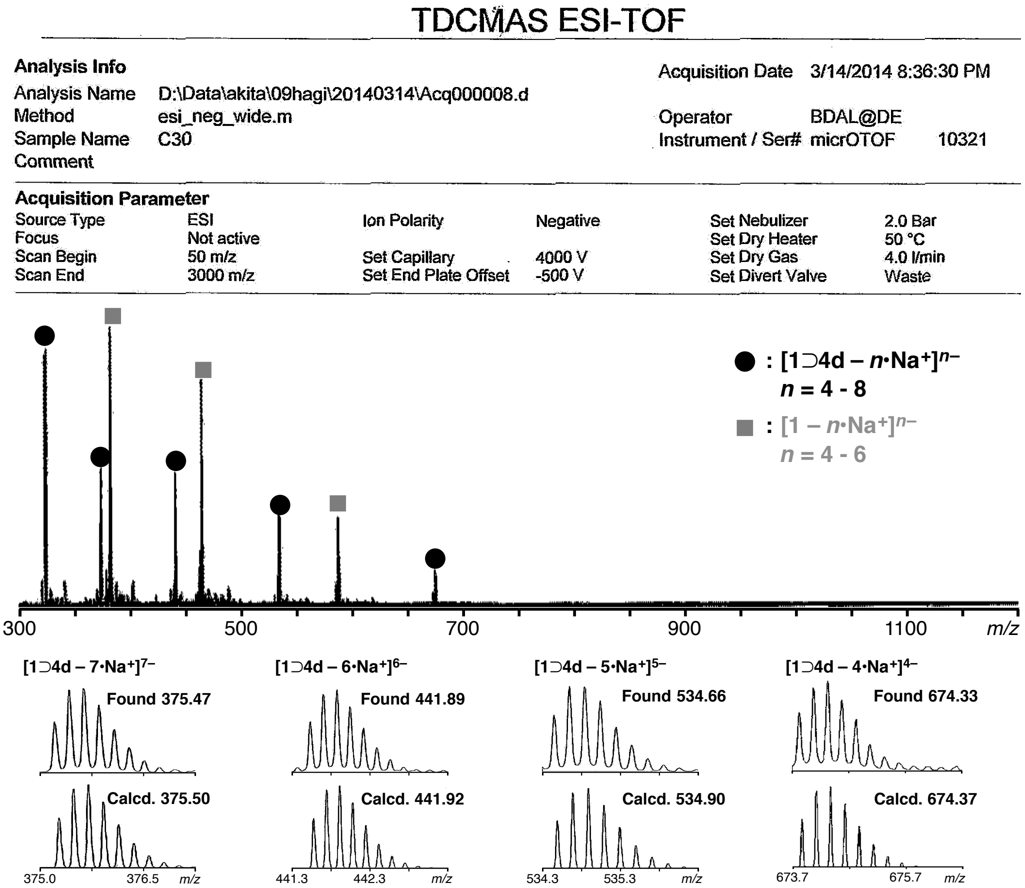

Figure S35. ESI-TOF MS spectrum (H_2O) of **1⊃4d**.

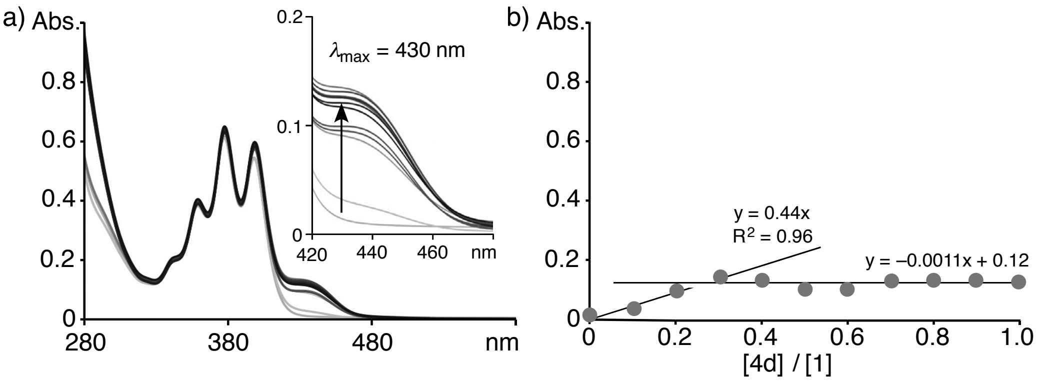

Figure S36. (a) Titration UV-vis spectra (0.2 mM, H_2O, r.t.) and (b) the plot (λ_{abs} = 430 nm) of **1** by the addition of **4d** ([**4d**]/[**1**] = 0.1, 0.2, 0.3, 0.4, 0.5, 0.6, 0.7, 0.8, 0.9, 1.0).

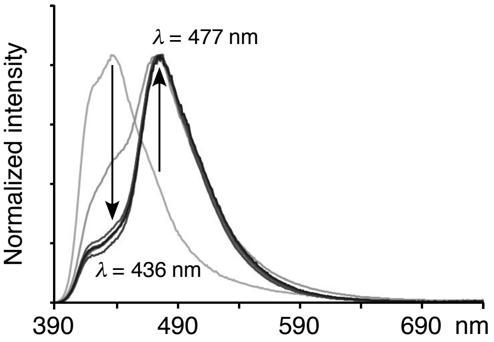

Figure S37. Titration fluorescence spectra (λ_{ex} = 378 nm, 0.2 mM, H_2O, r.t.) of **1** by the addition of **4d** ([**4d**]/[**1**] = 0.1, 0.2, 0.3, 0.4, 0.5).

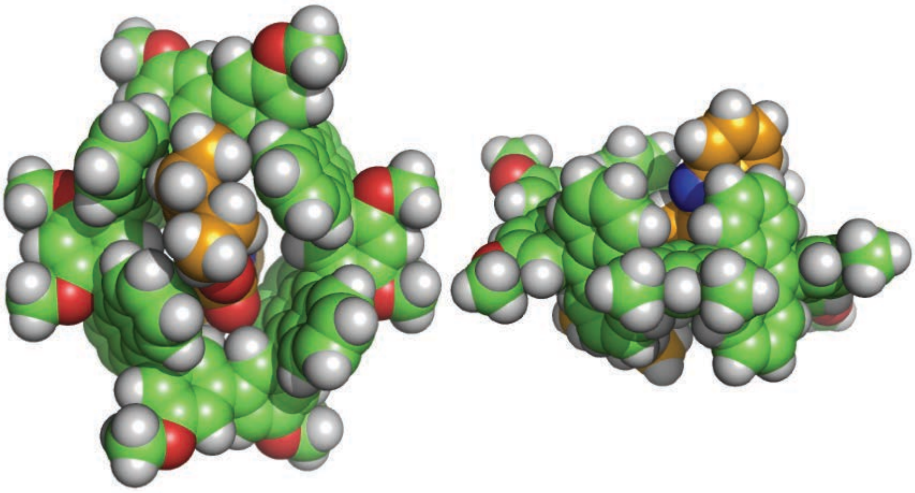

Figure S38. Optimized structure of a **1**⊃**4d** complex.

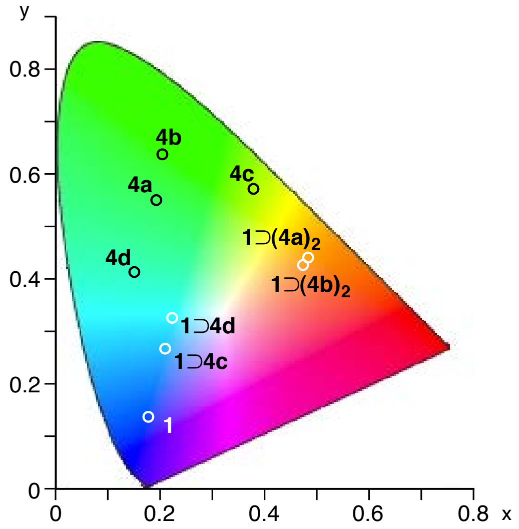

Figure S39. CIE chromaticity diagram (λ_{ex} = 378 nm, 0.2 mM, H_2O, r.t.) of **1**, **4a-d**, **1**⊃(**4a**)$_2$, **1**⊃(**4b**)$_2$, **1**⊃**4c**, and **1**⊃**4d**.

Competitive binding experiment of coumarin guests KH-361

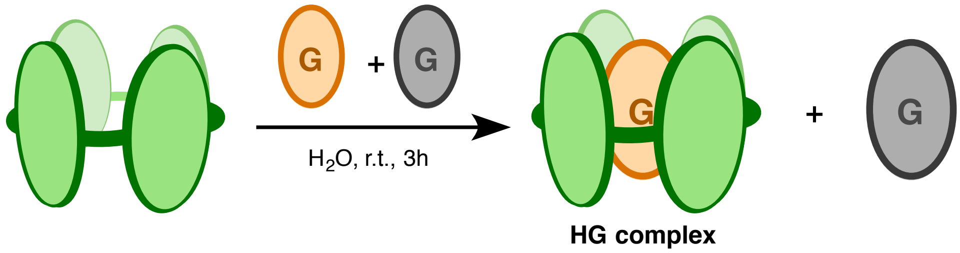

Coumarin dyes **4a** and **4b** (1.0 μmol each) were added to an H_2O solution (0.5 mL) of tube **1b** (0.025 mg, 0.10 μmol) in a glass test tube. The solution was stirred at r.t. for 3 h. After filtration, the formation of host-guest complexes was confirmed by UV-vis analysis. Similarly, competitive binding experiments of coumarin guests, **4a** vs. **4c**, **4a** vs. **4d**, **4b** vs. **4c**, and **4b** vs. **4d** were examined.

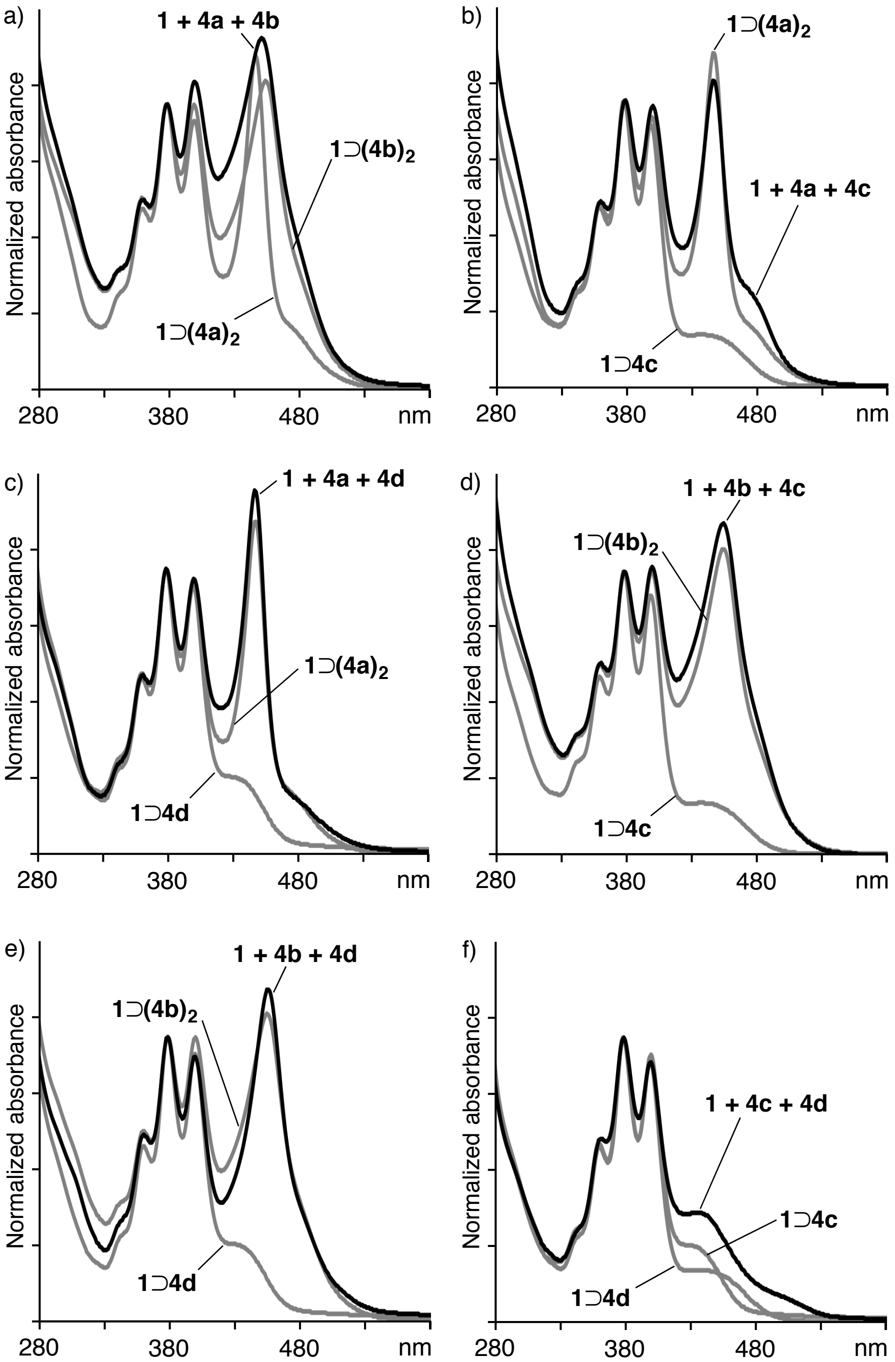

Figure S40. Fluorescent spectra (λ_{ex} = 378 nm, 0.2 mM, H_2O, r.t.) of competitive binding experiments after mixing (a) **1+4a+4b**, (b) **1+4a+4c**, (c) **1+4a+4d**, (d) **1+4b+4c**, (e) **1+4b+4d**, and (f) **1+4c+4d** in H_2O for 3 h at r.t.
